# A Personal Scientific Journey in Ophthalmology: Twenty-Five Years of Translating Research into Novel Therapies

**DOI:** 10.3390/ph18060883

**Published:** 2025-06-12

**Authors:** Dario Rusciano

**Affiliations:** Fidia Ophthalmic Research, 95123 Catania, Italy; drusciano55@gmail.com

**Keywords:** ophthalmic pathologies, neuroprotection, glaucoma, retinopathies, dry eye syndrome, nutraceuticals, drug delivery systems, visual rehabilitation, optogenetics, multisensory integration

## Abstract

Ocular diseases including glaucoma, diabetic retinopathy and age-related macular degeneration represent a growing global health burden, with current treatments often providing only symptomatic relief. Through an integrated approach combining preclinical models, molecular biology, and clinical insights, this review synthesizes 25 years of my translational research to advance therapeutic strategies for these conditions. Key findings demonstrate the following: (1) the dual neuroprotective and intraocular pressure-lowering effects of natural compounds (EGCG, forskolin) in glaucoma models; (2) successful development of Uparant, a first-in-class peptide inhibitor of pathological angiogenesis with efficacy in retinal disease models; and (3) innovative drug delivery systems (melatonin nanomicelles, liposomal sprays) that enhance ocular bioavailability. Notably, some of these approaches have progressed to early-phase clinical trials, demonstrating translational potential. Significant challenges remain in optimizing sustained drug delivery and addressing the heterogeneity of ocular diseases through personalized approaches. Future directions include combinatorial therapies and the application of artificial intelligence for treatment optimization. Collectively, this work establishes a framework for developing multi-target therapies that address both the molecular mechanisms and clinical needs in ophthalmology.

## 1. The Limitations of Current Ophthalmic Therapies

The development of truly disease-modifying therapies for ophthalmic conditions remains one of the most pressing challenges in vision science. While anatomical compartmentalization (anterior vs. posterior segment) and disease heterogeneity (e.g., dry eye subtypes, glaucomatous neurodegeneration patterns) demand tailored approaches, most clinical interventions still target symptoms rather than the underlying mechanisms. This therapeutic plateau stems from three fundamental barriers: (1) physiological obstacles to drug delivery, (2) an incomplete understanding of disease progression cascades, and (3) insufficient integration between mechanistic research and clinical translation.

The major ophthalmic diseases, including glaucoma, macular degeneration, dry eye syndrome, and diabetic retinopathy, present significant challenges to the global healthcare systems. As reported by the World Health Organization (WHO) in 2023, over 2.2 billion individuals worldwide are affected by vision impairments, with nearly half of these cases being either untreated or preventable [1]. This global health challenge imposes a significant economic toll, with an estimated annual loss of USD 411 billion in productivity [2,3]. In the United States, vision problems are particularly prevalent. Recent statistics indicate that roughly 90 million Americans aged 40 and older—equating to more than 60% of this age group—are affected by visual impairments or ocular disorders [2,3]. Despite considerable advancements in medical science, these conditions often remain difficult to treat effectively, with existing therapies frequently addressing only the symptoms rather than offering definitive cures [4]. This inadequacy underscores the pressing need to explore additional approaches that can complement standard treatments and enhance their efficacy.

Current ophthalmic therapies range from pharmacological interventions to surgical procedures, and they have undoubtedly improved outcomes for many patients. For example, anti-vascular endothelial growth factor (anti-VEGF) injections have revolutionized the management of neovascular age-related macular degeneration (AMD) [5], and prostaglandin analogs and beta-blockers are foundational in glaucoma therapy [6]. However, these treatments are not without limitations in safety and/or efficacy. One of the primary challenges in ophthalmology is the effective delivery of drugs to the eye. The eye’s unique structure, including the presence of barriers such as the cornea, sclera, and the blood–retina barrier, often restricts the penetration and efficacy of medications [7]. This physiological barrier necessitates the development of more advanced drug delivery systems to ensure that therapeutic agents reach their intended targets in adequate concentrations [8]. Moreover, many ophthalmic drugs come with side effects and adverse reactions. For instance, patients using anti-VEGF injections may experience ocular discomfort, endophthalmitis, and increased intraocular pressure [9]. Similarly, prostaglandin analogs used in glaucoma therapy can cause hyperemia, eyelash growth, and changes in iris pigmentation [10]. These side effects can complicate the management of the diseases and reduce patient adherence to the prescribed treatments.

Another significant restriction is the limited duration of the effect offered by many treatments. Anti-VEGF injections, for example, require frequent administration—often on a monthly basis. This frequent dosing regimen can be burdensome for patients, increasing the risk of complications, such as infections, and adding to the overall cost and logistical challenges of treatment [11].

Non-responsiveness is another critical issue. A subset of patients does not respond adequately to standard treatments, necessitating the development of alternative therapeutic strategies [12]. This variability in treatment responses highlights the need for personalized medicine approaches that tailor therapies to individual patient profiles based on genetic and clinical factors. Furthermore, the long-term use of certain medications can impact patients’ quality of life. The chronic administration of eye drops for conditions such as glaucoma can lead to ocular surface disease, which includes symptoms like dryness, irritation, and visual disturbance [13]. The need for the daily administration of these medications can also be a constant reminder of the disease, affecting patients’ psychological well-being [14].

Regulatory and manufacturing challenges play an essential but critical role in limiting the development of new ophthalmic drugs and delivery systems. The stringent regulatory environment ensures the safety and efficacy of new therapies, but it also creates hurdles that can delay the introduction of innovative treatments. Additionally, the complexity of manufacturing advanced drug delivery systems, such as implants or nanoparticles, poses significant technical and economic challenges [15].

To address these limitations, researchers are exploring several innovative approaches. Advanced drug delivery systems, such as biodegradable implants, nanoparticles, and contact lenses, are being investigated to provide the sustained and targeted delivery of therapeutic agents [16]. These systems aim to overcome the anatomical barriers of the eye and reduce the frequency of administration, thereby improving patient adherence and outcomes. Gene and cell therapies represent another promising avenue, aiming to treat the underlying causes of ophthalmic diseases rather than merely managing symptoms. These therapies have the potential to offer long-lasting, if not permanent, solutions by addressing the genetic and cellular dysfunctions at the root of the diseases [17,18]. Combination therapies, which involve using multiple therapeutic agents or modalities, are also being explored to enhance efficacy and reduce side effects. By targeting different aspects of the disease process, combination therapies can provide a more comprehensive approach to treatment [19]. Finally, the field of personalized medicine is gaining traction in ophthalmology. By tailoring treatments to individual patients based on genetic, molecular, and clinical data, personalized medicine approaches can optimize therapeutic outcomes and minimize adverse effects [20].

Therefore, addressing all the above limitations through innovative research and development is essential for advancing the treatment of ophthalmic diseases and improving the quality of life for patients worldwide.

Beyond mechanical and pharmacological interventions, emerging research underscores the critical influence of hormonal imbalances in ocular diseases, offering another layer of complexity to their pathophysiology and treatment. In 2016, we delineated how sex steroids (e.g., estrogens, androgens), insulin, thyroid hormones, and melatonin modulate ocular tissue physiology, impacting conditions ranging from dry eye syndrome (DES) to glaucoma and age-related macular degeneration (AMD) [21]. For instance, sex steroid imbalances—particularly in postmenopausal women—correlate with DES severity due to their regulatory role in lacrimal and Meibomian gland function. Similarly, melatonin demonstrates dual utility in glaucoma, both as an intraocular pressure (IOP) modulator (via MT3 receptors in the ciliary body) and a neuroprotectant against retinal ganglion cell apoptosis. These findings highlight the potential for hormone-based chronotherapies and personalized approaches, particularly in diseases like glaucoma where circadian IOP fluctuations are clinically significant. However, while hormonal pathways offer promising adjunctive strategies, their integration into mainstream ophthalmic practice requires further mechanistic and clinical validation.

Based on the premises above, this review delves into several innovative treatments that I have contributed to developing and refining over the past 22 years of dedicated research in the field of ophthalmology, as documented by the 76 publications that I have authored or co-authored and referenced herein. My work has spanned a wide spectrum of ocular conditions, addressing both the anterior segment and retinal diseases. In the anterior segment, I have explored groundbreaking therapies for conditions such as glaucoma, dry eye syndrome, myopia, and keratoconus, focusing on novel pharmacological approaches and advanced drug delivery systems. In the realm of retinal diseases, my research has concentrated on pioneering treatments for age-related macular degeneration, diabetic retinopathy, and other retinal conditions. These efforts have included the development of targeted therapies and innovative surgical techniques and the integration of cutting-edge technologies to enhance therapeutic outcomes. Through this comprehensive review, I aim to present the key advancements and contributions that have emerged from my extensive research journey, highlighting the potential and impact of these innovative treatments in improving eye health and patient quality of life.

## 2. Ocular Surface and the Anterior Segment

The ocular surface, a complex and delicate structure, includes the cornea, conjunctiva, tear film, and associated glands. It serves as the eye’s first line of defense against environmental challenges and plays a vital role in maintaining visual clarity. However, this intricate system is vulnerable to a range of diseases that can significantly impair quality of life and visual function [22]. Additionally, the ocular surface is protected by a robust epithelial barrier, which, while essential for preventing the entry of pathogens and harmful substances, poses a significant challenge for the effective delivery of therapeutic agents. This barrier limits the penetration of drugs and treatments to the cornea and anterior segment, complicating the management of ocular surface diseases and necessitating innovative approaches to enhance therapeutic efficacy [7].

### 2.1. The Epithelial Barrier

The surface of the eye is covered by two types of epithelial cells: corneal and conjunctival cells. These cells play a crucial role in protecting the inner structures of the eye from physical, chemical, and biological aggression. The corneal epithelial cells cover the cornea, while the conjunctival epithelial cells cover the conjunctiva. These epithelial cells are strongly connected to one another through tight junctions, creating a robust physical barrier that helps maintain the integrity of the eye’s surface and prevents harmful substances from penetrating deeper into the eye [23]. Corneal epithelial cells are responsible for maintaining corneal transparency and providing a smooth refractive surface—critical for the cornea’s role as the eye’s primary lens—while forming a tight barrier against pathogens and fluid loss.

Recent investigations into the implications of COVID-19 have illuminated the ocular involvement of SARS-CoV-2, underscoring the eyes not just as a potential entry point for the virus but as a site of viral presence itself. Accordingly, we conducted a study on a cohort of 74 COVID-19-positive Polish patients, revealing that the virus could be detected in ocular swabs, albeit infrequently, with a positive rate of 4.05% [24]. This study’s findings indicate that while overt ocular infection is rare, more than 47% of patients exhibited ocular symptoms, emphasizing the need for heightened awareness of ocular manifestations in COVID-19 cases. We noted a lack of correlation between the severity of respiratory symptoms and ocular findings, suggesting that ocular symptoms could occur independently of respiratory distress—a critical factor for ophthalmologists and healthcare providers. Additionally, we proposed that ocular surfaces could act as a possible vector for viral transmission, highlighting the importance of protective measures for healthcare professionals working with patients exhibiting ocular symptoms during the pandemic.

The corneal epithelium is composed of multiple layers of cells, including superficial flat cells, wing cells, and basal columnar cells. The basal cells are capable of rapid mitosis in response to injury and represent ‘transient amplifying cells’. The corneal epithelium is anchored to the underlying Bowman’s layer through hemidesmosomes and anchoring filaments [23]. The cornea’s avascularity is critical for transparency but complicates repair after injury. Alkali burns, for instance, disrupt this balance, triggering pathological neovascularization. As we demonstrated in a rabbit model, alkali burns induced neovessel formation in the corneal stroma within 3 weeks, escalating by 6 weeks, highlighting the cornea’s compromised barrier function and the role of inflammatory mediators in driving angiogenesis [25]. This aligns with clinical observations where corneal vascularization exacerbates opacity and visual impairment [26].

**Figure 1:**
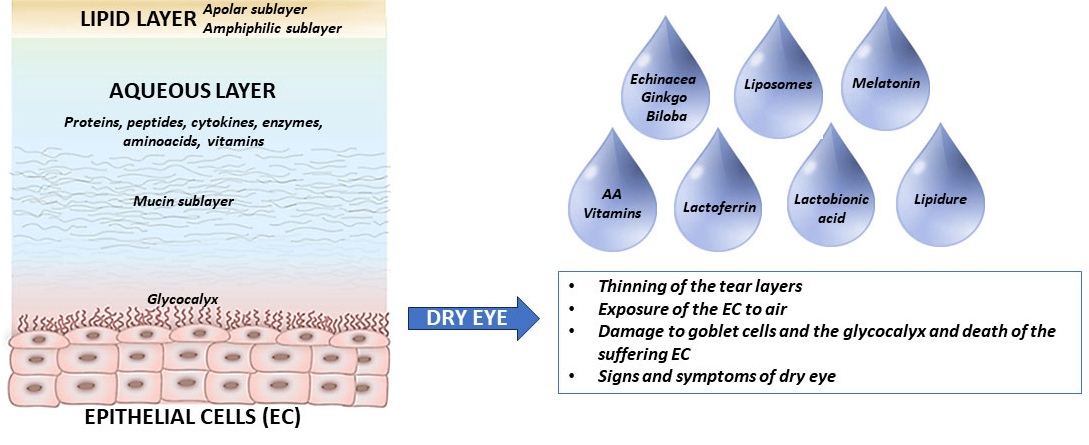
Alterations of the tear film layer in dry eye. The left panel illustrates the normal layered structure of a healthy tear film. The right panel shows pathological changes characteristic of dry eye and some of the potential therapeutic molecules for eye drop formulations.

The conjunctival epithelium is multilayered near the lid margins but about 2–3-cells thick elsewhere. Goblet cells within the conjunctival epithelium produce mucins, which form the innermost layer of the tear film [23] (Figure 1). In this way, they contribute to the immune defense by providing a protective layer against environmental irritants.

#### 2.1.1. Bovine Conjunctival Epithelial Cells (BCEC)

The isolation, characterization, and in vitro expansion of ocular surface epithelial cells are critical for investigating disease mechanisms and developing preventive or therapeutic strategies for ocular surface pathologies. These processes are essential for investigating the cellular mechanisms underlying ocular surface diseases, including dry eye syndrome, corneal ulcers, and inflammatory disorders. The isolation and characterization of these cells enable the identification of disease-specific markers and pathogenic pathways. In vitro culture systems facilitate the evaluation of potential therapeutic interventions—such as pharmacological agents and regenerative therapies—allowing for efficacy and safety assessments prior to clinical translation. With this goal in mind, we established and used different cell culture techniques to investigate cytokeratin expression in primary epithelial cell cultures derived from bovine conjunctiva, focusing on maintaining the conjunctival phenotype during in vitro culture [27]. By employing both liquid-covered and air-interface culture conditions, we monitored the expression of key cytokeratins (CK4, CK13, CK19) specific to the conjunctival epithelium, alongside physiological parameters like the trans-epithelial electrical resistance (TEER), which measures the barrier properties of the multilayer. The findings demonstrate that conjunctival epithelial cells retain their characteristic phenotype under air-interface conditions, with no transition to a corneal phenotype. This model provides a reliable in vitro surrogate for conjunctival tissue, enabling pharmacological studies and advancing research on ocular surface diseases, particularly regarding epithelial stability, drug testing, and potential therapeutic interventions.

Using these model systems, we investigated the role of lactoferrin (LF) on the ocular surface and established a primary bovine conjunctival epithelial cell (BCEC) culture system as a model to study LF regulation [28]. Lactoferrin, a key tear film component secreted predominantly by the lacrimal glands, plays a vital role in ocular surface protection through its antimicrobial, anti-inflammatory, and iron-binding activities [29]. Our study demonstrated that both the corneal and conjunctival epithelia produce LF, with conjunctival tissues exhibiting higher levels of expression. Using RT-PCR, Western blotting, and promoter transfection assays, we showed that BCECs retain their epithelial characteristics in vitro, produce lactoferrin, and support the expression of a reporter gene driven by the human LF promoter. Importantly, promoter activity peaked in second-passage (P2) cultures and declined in later passages, suggesting temporal variations in cellular stability during culture. The model is particularly relevant for studying regulatory mechanisms that govern LF expression, offering insights into how transcription factors, like Sp1 or estrogen receptors, and environmental factors, such as retinoic acid, may influence LF production. These findings are clinically significant, as reduced LF levels are associated with conditions like dry eye syndrome and Sjögren’s syndrome, where increased LF production could restore tear film stability and protect the ocular surface. By enabling the identification of factors that induce endogenous LF expression, this model system provides a promising platform for developing novel therapies for ocular surface disorders.

#### 2.1.2. The Statens Seruminstitut Rabbit Cornea (SIRC) Cell Line

The SIRC cell line is often used to study the physiology and pathology of ocular surface diseases. It is derived from rabbit corneal epithelial cells and is commonly used in research related to corneal infections, inflammation, and wound healing. The SIRC cell line provides a relevant in vitro model for investigating various aspects of ocular surface biology and disease mechanisms [30].

We used the SIRC cell line to evaluate the cytotoxicity and antimicrobial efficacy of a novel eye drop preservative formulation combining N-hydroxymethylglycinate (NIG) and EDTA [31]. The study compared this formulation to commonly used preservatives such as benzalkonium chloride (BAK), polyquaternium-1 (PQ-1), and sodium perborate (SP). The findings revealed that NIG–EDTA demonstrated significantly lower cytotoxicity against this cell line while maintaining effective antimicrobial properties at concentrations typically used in ophthalmic solutions. The relevance of this study lies in addressing the well-documented adverse effects of traditional preservatives, such as corneal cell apoptosis, tear film instability, and inflammation, which are particularly concerning for patients requiring chronic eye drop usage, such as those with glaucoma or dry eye syndrome. The NIG–EDTA formulation showed no significant cytotoxicity even after prolonged exposure (up to 48 h) and, intriguingly, promoted cell growth under certain conditions due to the calcium-chelating properties of EDTA. This feature may further enhance the tolerability of the formulation on the ocular surface. For researchers studying in vitro treatments for ocular surface diseases, this study highlights the importance of selecting low-toxicity preservatives to minimize cellular damage while maintaining sterility. NIG–EDTA offers a promising alternative to traditional preservatives and could improve the safety profile of multi-dose ophthalmic formulations, making it a key consideration in the development of eye drops for both therapeutic and long-term maintenance use.

In a later seminal study, also published in 2018 [32], we addressed the molecular and phenotypic characteristics of the SIRC cell line. Our study demonstrated that SIRC cells exhibit a hybrid nature, expressing markers of both epithelial and fibroblastic lineages. Specifically, these cells express epithelial markers such as cytokeratins CK3/CK12 and tight junction proteins (occludin and claudins), albeit at lower levels compared to classical epithelial cell lines like ARPE-19. Simultaneously, SIRC cells also express fibroblastic markers such as vimentin, lumican, and α-SMA, indicating a shift toward a mesenchymal phenotype. The hybrid nature of SIRC cells makes them a relevant model system for evaluating the efficacy and toxicity of treatments targeting ocular surface diseases. This cell line can mimic the characteristics of both corneal epithelial and stromal cells, making it versatile for studying drug permeability, wound healing, and epithelial-to-mesenchymal transition (EMT) processes. Furthermore, the study highlighted the impact of long-term in vitro culture on the phenotypic drift of cell lines, which is a critical consideration for reproducibility and the interpretation of results in preclinical research. By acknowledging these characteristics, the SIRC cell line emerged as a valuable yet complex tool for assessing novel therapeutics, including their effects on the corneal epithelium and their potential to modulate fibroblast-driven stromal remodeling.

#### 2.1.3. Human Corneal Epithelial Cells (HCE-F)

Next, we set out to develop a human corneal epithelial cell line (HCE-F) from a donor cornea, aiming to provide a reliable in vitro model for studying the biological responses of the corneal epithelium to various stressors and therapeutic treatments [33]. By treating a corneal button with enzymes to separate the epithelial cells, we successfully cultured these cells, which exhibited a classical polygonal morphology and maintained this characteristic over multiple in vitro passages. The HCE-F cells demonstrated typical barrier-forming properties, confirmed through TEER measurements, and expressed cytokeratins consistent with corneal epithelial cells, predominantly CK3 and CK5. A karyotype analysis revealed the presence of two trisomies, in chromosomes 8 and 11. Concluding that HCE-F is a stable and true primary human corneal epithelial cell line, the study suggests its potential utility as a test cell line for examining the biological response of the human corneal epithelium to both stress and therapeutic interventions and for comparisons with epithelial cells derived from pathological corneas. This makes the HCE-F cell line particularly relevant for studying the physiology and pathology of ocular surface diseases, providing a valuable tool for advancing understanding and treatment in this field.

#### 2.1.4. Human Keratocytes and Myofibroblast Transition

Using a commercially available human keratocyte cell line, we investigated the combined effects of vitamins A and E with hyaluronic and lactobionic acids on preventing the transformation of keratocytes into myofibroblasts [34]. This transition is a significant contributor to corneal haze and scarring, particularly after ocular surgeries like photorefractive keratectomy (PRK). Myofibroblasts are responsible for the excessive deposition of extracellular matrix, leading to fibrosis and reduced corneal transparency. This study demonstrates that the combination of these vitamins with hyaluronic and lactobionic acids provides a protective effect against the molecular changes that drive this transition. Vitamins A and E are known for their antioxidant properties, which help reduce oxidative stress, a key factor in inflammation and fibrosis. Hyaluronic acid contributes to the maintenance of hydration and lubrication in the corneal tissue, while lactobionic acid offers additional antioxidant support and enhances the bioavailability of the vitamins. By preventing the keratocyte-to-myofibroblast transition, this combination treatment could significantly reduce post-surgical inflammation and promote better healing outcomes. This approach offers a safer alternative to conventional treatments that may have higher toxicity levels. Additionally, the therapy’s anti-inflammatory and tissue-preserving effects make it relevant for managing other inflammatory conditions of the anterior segment, beyond post-surgical contexts. Overall, this study provides valuable insights into potential non-toxic therapeutic strategies for controlling inflammation and promoting healthy corneal repair, highlighting its importance in the field of ophthalmology.

### 2.2. Eye Color

The desire to change eye color arises from both aesthetic and medical motivations, with individuals seeking alterations for cosmetic enhancement or to address medical conditions such as corneal opacities, albinism, or aniridia. Several methods are available to achieve this goal, each with varying degrees of invasiveness and risk [35]. Colored contact lenses offer a non-invasive and reversible solution, allowing individuals to temporarily alter their eye color. However, improper fitting or maintenance can lead to corneal irritation, discomfort, or infection. More permanent approaches include laser eye color change, which uses low-energy lasers to reduce melanin in the iris, effectively turning brown eyes blue. While this method is relatively quick and permanent, it remains experimental and poses risks such as increased intraocular pressure and uveitis. Iris implant surgery, originally developed to treat conditions like heterochromia or traumatic iris damage, involves placing a synthetic implant over the iris. Although this method offers a permanent solution, it is highly invasive and carries significant risks, including glaucoma, cataracts, corneal damage, and vision loss, making it controversial for cosmetic purposes [36]. Another option is keratopigmentation, a surgical procedure in which pigment is applied or tattooed into the corneal stroma to alter the eye color. Initially developed to mask corneal scars or opacities, keratopigmentation has gained popularity for cosmetic purposes. This method is less invasive than iris implants and generally safer, but complications such as pigment diffusion, irregular coloration, or infection may occur [37]. Additionally, certain medications, such as prostaglandin analogs used for glaucoma treatment, can darken the iris over time by increasing melanin production in stromal melanocytes, but this effect is irreversible and limited to darkening brown or hazel eyes. While contact lenses remain the safest option for cosmetic eye color changes, methods such as keratopigmentation, laser procedures, and iris implants require careful consideration due to their associated risks.

We used strongly pigmented melanoma cell lines—B16-F1 (a mouse cutaneous melanoma) and 92.1 (a human uveal melanoma)—as test models to investigate whether certain natural, non-toxic biological compounds could reversibly inhibit melanin production, thereby offering a safe and temporary method to alter eye color [38]. This study explores the potential of argan oil as an innovative and safe approach to modulate melanogenesis in human uveal melanoma cells, with implications for both cosmetic and medical applications. By investigating its effects on 92.1 uveal melanoma cells, we demonstrated that argan oil significantly reduces melanin production and tyrosinase expression (the enzyme responsible of melanin production) in a time-dependent manner, without causing cytotoxicity. This depigmenting effect is attributed to the downregulation of key signaling pathways, including ERK1/2 and PI3K/Akt, as well as the phosphorylation and degradation of the melanogenesis-associated transcription factor MITF. The study highlights that the molecular mechanisms regulating melanogenesis in uveal cells differ from those in cutaneous cells, underscoring the need for specialized approaches in ocular pigmentation research. The findings suggest that argan oil could serve as a non-toxic, reversible modulator of melanin production, presenting a novel avenue for the cosmetic alteration of iris color or the treatment of hyperpigmentation-related ocular conditions. This safe and effective method addresses the growing demand for natural alternatives in cosmetic applications, while offering insights into the broader regulation of ocular melanogenesis.

### 2.3. Dry Eye

Dry eye disease (DED) is a widely prevalent and often underdiagnosed condition that affects millions of people worldwide, with its incidence increasing due to aging populations and lifestyle factors such as prolonged digital screen use. Characterized by a dysfunctional tear film, it can lead to ocular surface damage, irritation, and chronic inflammation, significantly impairing quality of life (Figure 1). Dry eye is not only a source of constant discomfort but can also be deeply invalidating, with symptoms ranging from mild dryness and itching to severe pain, visual disturbances, and sensitivity to light. Its multifactorial nature includes metabolic causes like diabetes, hormonal imbalances, and autoimmune disorders (e.g., Sjögren’s syndrome), as well as infective and inflammatory processes that disrupt the homeostasis of the tear film and ocular surface [39]. Understanding these diverse etiologies is essential for developing targeted treatments to alleviate the burden of this pervasive condition.

We have investigated various approaches, both systemic and topical, to treat and alleviate the signs and symptoms of dry eye.

#### 2.3.1. Amino Acids

Amino acids, the fundamental building blocks of proteins, play a crucial role in maintaining the health of the ocular surface. Human tears naturally contain 23 amino acids, which contribute to the stability and function of the tear film. In individuals with dry eye disease (DED), the balance of these amino acids can be disrupted, leading to ocular surface damage and discomfort [40]. To address this imbalance, we have explored both topical eye drops and oral supplements enriched with specific amino acids as therapeutic options [41]. The incorporation of amino acids into treatments for DED is based on their ability to support various physiological processes essential for ocular surface health. Amino acids contribute to the repair of damaged epithelial cells and protect against oxidative stress [42], which is heightened in DED. Certain amino acids (including glycine, proline, isoleucine, leucine, phenylalanine, valine, β-alanine, taurine) function as osmoprotectants, helping cells to maintain their volume and integrity under hyperosmolar conditions [43], commonly seen in dry eye patients. Some amino acids exhibit anti-inflammatory properties, reducing the ocular surface inflammation associated with DED [41].

Two seminal studies by the lab of Vinciguerra highlighted the significant and multifaceted role of amino acids in corneal healing post-surgery. In the first study [44], researchers found that corneas incubated with amino acids showed markedly improved re-epithelialization and organized healing compared to those in standard storage media. This enhanced healing process is crucial for maintaining corneal transparency and function, thereby improving patient outcomes after refractive surgeries such as PRK. The second study from the same lab focused on the systemic benefits of amino acid supplementation [45]. It demonstrated that the oral intake of amino acids significantly promoted corneal nerve regrowth and density following PRK. Corneal nerves are essential for ocular surface health, as they help regulate tear production and provide sensory feedback. Improved corneal nerve regeneration not only aids in faster recovery but also reduces the risk of complications such as neurotrophic keratitis and persistent epithelial defects.

A later study by Roszkowska and colleagues [46] further supported the role of amino acids in corneal healing post-surgery. This study investigated the effects of oral amino acid supplementation on corneal nerve regrowth after PRK. The researchers divided 40 patients into two groups: one received oral amino acid supplementation before and after surgery, while the other group did not. Using in vivo corneal confocal microscopy, they evaluated sub-basal corneal nerve fibers over 12 months. The results showed significantly faster nerve regeneration and higher nerve fiber density in the supplemented group compared to the control group. This study highlights the potential of oral amino acid supplementation as an additional treatment to enhance corneal nerve restoration and improve overall ocular surface health after refractive surgery.

Together, these studies underscore the metabolic importance of complex amino acid mixtures given systemically in restoring ocular surface homeostasis after surgery. By supporting both epithelial repair and nerve regeneration, amino acids contribute to a more comprehensive healing process. This dual role makes them an invaluable component of post-surgical care for patients undergoing refractive procedures, helping to reduce inflammation, enhance recovery, and ensure better long-term visual outcomes. Research highlighted in a review by Pellegrini et al. [47] discusses the potential benefits of various nutrients, including amino acids, for ocular surface health. The review suggests that systemic supplementation may support tear film stability and ocular surface integrity, offering a complementary approach to topical treatments.

Considering topical applications, four amino acids were chosen to be added to eye drops for dry eye: proline, lysine, leucine, and glycine. The rationale for incorporating such amino acids into eye drops for the treatment of dry eye and other ocular surface diseases lies in their specific biochemical properties and physiological roles that directly benefit the ocular surface. These amino acids are essential for maintaining cellular health, promoting tissue repair, and mitigating inflammation, all of which are critical for managing the symptoms and damage associated with dry eye disease (DED) [41]. Proline plays a vital role in the synthesis of collagen, a structural protein essential for maintaining the integrity of the extracellular matrix and promoting corneal wound healing. It acts as a stabilizer for protein structures and can help strengthen the corneal epithelium, which is often compromised in dry eye conditions. Proline also contributes to osmoprotection, enabling cells to adapt to hyperosmolar stress, a hallmark of dry eye disease. Lysine is a crucial amino acid involved in protein synthesis and the cross-linking of collagen fibers. This property is particularly important for reinforcing the structural stability of the corneal tissue. Lysine also enhances the bioavailability of other nutrients and contributes to the repair of damaged epithelial cells, promoting faster recovery of the ocular surface. Its anti-inflammatory properties further support its role in reducing the chronic inflammation characteristic of dry eye. Leucine, a branched-chain amino acid, is essential for cellular metabolism and tissue regeneration. It serves as a key regulator of mTOR signaling, which controls cell growth and protein synthesis. In the context of dry eye, leucine supports the repair and proliferation of epithelial cells, helping restore the damaged corneal barrier. Its role in maintaining cellular energy and reducing oxidative stress is particularly beneficial for alleviating the damaging effects of inflammation and hyperosmolarity. Glycine, the simplest amino acid, has well-established anti-inflammatory and cytoprotective properties. It is a precursor for the synthesis of glutathione, a critical antioxidant that protects the ocular surface from oxidative damage caused by reactive oxygen species (ROS). Glycine also stabilizes cell membranes and promotes osmoprotection, which is crucial for maintaining tear film stability and preventing desiccation of the ocular surface. By incorporating these amino acids into eye drop formulations, the treatment leverages their collective benefits to address the multifactorial nature of dry eye disease. They support the healing of epithelial damage, enhance tear film stability, mitigate inflammation, and protect the ocular surface from oxidative stress and hyperosmolarity. This targeted approach not only alleviates symptoms but also addresses the underlying causes of ocular surface dysfunction, offering a comprehensive solution for managing dry eye and other related conditions [41].

A clinical study by Aragona et al. [48] evaluated the therapeutic potential of amino-acid-enriched tear substitutes for individuals suffering from dysfunctional tear syndrome (DTS), a condition characterized by an unstable tear film and ocular surface damage. The study involved a clinical trial with DTS patients, who were treated with eye drops containing a combination of the four amino acids. Over the course of the treatment, the researchers assessed the corneal surface health using various clinical parameters, including the tear film break-up time (BUT), fluorescein staining, and patient-reported symptoms. The results demonstrated that patients using the amino-acid-enriched tear substitutes experienced significant improvements in both objective clinical measures and subjective symptoms of dry eye. Specifically, the presence of amino acids in the eye drops helped to enhance the regeneration and repair of the corneal epithelium, leading to a more stable tear film and reduced ocular surface damage. The study concluded that amino acids play a crucial role in maintaining ocular surface homeostasis by providing the necessary building blocks for protein synthesis and cellular repair processes. This makes amino-acid-enriched tear substitutes a promising therapeutic option for managing DTS and improving the quality of life for affected patients. By highlighting the importance of amino acids in promoting corneal healing and maintaining the ocular surface integrity, this study adds valuable insights to the growing body of evidence supporting the use of amino acids in the treatment of ocular surface diseases.

In summary, the supplementation of amino acids through eye drops or oral intake presents a promising avenue for the management of dry eye disease. By addressing the underlying deficiencies and supporting the natural physiology of the tear film and ocular surface, amino-acid-based therapies offer a targeted approach to alleviate the symptoms and progression of DED.

#### 2.3.2. Liposomes Spray (Lacrisek^TM^)

Evaporative dry eye is a prevalent condition characterized by a deficiency in the lipid layer of the tear film, leading to excessive evaporation and discomfort. Liposomal sprays have emerged as a promising treatment by delivering phospholipid liposomes directly to the tear film. The use of liposomes targets the lipid layer deficiency that affects about 80% of dry eye sufferers [49]. We at Sooft have developed new liposomal formulations enriched with amino acids and vitamins, with the aim of further enhancing the therapeutic options available for managing dry eye disease (DED) [50].

The liposomal spray is applied to the closed eyelid, allowing the liposomes to migrate to the tear film. This application technique increases lipid layer thickness and improves tear film stability. The inclusion of amino acids and vitamins in newer formulations like Lacrisek Ofta Plus^®^ offers additional benefits, potentially enhancing the overall healing properties of the tear substitute.

Clinical studies indicate that the use of phospholipid liposomes can help restore the lipid layer, thus reducing the evaporation rate and improving ocular surface health. A study by Craig et al. [51] found that the lipid layer grade (LLG) increased significantly in treated eyes compared to control eyes, indicating that liposomal application directly benefits the lipid layer of the tear film. Statistically significant improvements in lipid layer thickness and tear film stability were observed for over an hour after application. The non-invasive tear film break-up time (NIBUT) was improved significantly in eyes treated with the liposomal spray compared to saline controls, demonstrating enhanced stability of the tear film [51]. In a comparative study, Khaireddin and Schmidt [52] demonstrated that phthalate-liposomal eye spray outperformed hyaluronate artificial tears, showing greater improvement in lid-parallel conjunctival folds (LIPCOFs), a clinical marker of dry eye severity reflecting mechanical friction and conjunctival laxity. Subjective reports indicated that 68% of participants preferred the liposomal spray over the control, with significant improvements in comfort levels reported within 30 min of application [51]. In a comparative study by Fogagnolo et al. [53], the efficacy of two lipid-based eye drops—Lacrisek Ofta (liposomes) and Artelac Rebalance (PEG and hyaluronic acid)—was assessed in patients with moderate evaporative dry eye. The liposome-containing formulation showed quicker, deeper, and longer-lasting effects, improving the blink frequency, break-up time (BUT), and overall ocular comfort.

A pilot prospective study by Modugno et al. [50] tested Lacrisek Ofta Plus, the new liposomal tear substitute enriched with amino acids (L-proline, L-glycine, L-lysine hydrochloride, and L-leucine) and vitamins A and E, on patients with evaporative DED. Sixteen eyes were treated for 30 days, leading to significant improvements in the tear film break-up time (TF-BUT) and reduced fluorescein staining scores. Importantly, the incidence of elevated MMP-9 levels (as indicated by the Inflamma-Dry test) significantly decreased, suggesting the effective management of inflammation and symptom relief.

In conclusion, the combination of liposomal sprays with traditional therapies is suggested to represent a significant advancement more specifically in the treatment of evaporative dry eye.

#### 2.3.3. Lactoferrin and Lactobionic Acid (Lactoyal^TM^)

Lactoferrin (LF) is a glycoprotein present in various secretions, including tears, where it plays a vital role in protecting the ocular surface through its antimicrobial and anti-inflammatory properties. The presence of LF in the tear film helps maintain homeostasis, support epithelial cell integrity, and prevent microbial infections. Recognizing the importance of LF, we have investigated the feasibility of isolating the lactoferrin gene and transferring it into ocular surface epithelial cells to enhance the endogenous expression of LF [28]. We utilized primary bovine conjunctival epithelial cell (BCEC) cultures and assessed LF mRNA expression using quantitative real-time PCR and Western immunoblotting, confirming the capability of these cells to produce LF. Moreover, a transient transfection system was used to analyze the regulatory elements of the human lactoferrin promoter, suggesting that understanding the mechanisms of LF expression in ocular tissues could inform potential gene therapy strategies aimed at increasing LF levels on the ocular surface.

Meanwhile, research on lactobionic acid (LA), a disaccharide and lactose derivative, revealed its promising role as a functional substitute for LF. LA possesses multiple bioactive properties; it is hygroscopic, acts as a potent antioxidant, and inhibits matrix metalloproteinase (MMP) activity, contributing to its protective effects on the ocular surface [54]. In a preclinical study, we examined LA as an additive to artificial tears, demonstrating significant efficacy in promoting wound healing in both in vitro models using rabbit corneal cells and in vivo models with rabbit corneas subjected to epithelial debridement [32]. We reported that LA significantly reduced levels of MMP-9 and transforming growth factor beta (TGF-β), suggesting the effective management of inflammation and support for epithelial regeneration. Moreover, LA exhibited antibacterial properties by inhibiting the growth of *Staphylococcus aureus*, indicating its capability to mitigate infection risks. Complementing these preclinical findings, we also provided clinical evidence of LA’s effectiveness, demonstrating that a combination of hyaluronic acid (HA) and LA significantly improved dry eye symptoms in older patients, who often exhibit decreased tear lactoferrin levels [55]. This study highlighted the therapeutic potential of LA in mimicking the protective properties of lactoferrin, addressing the inflammatory, oxidative, and antimicrobial aspects of ocular surface disease. The study pointed out the potential for treatments based on LF or LA to mitigate dryness and inflammation, particularly in age-related dry eye conditions.

Collectively, these studies illustrate the potential of both lactoferrin enhancement strategies and lactobionic acid functional substitutes in advancing therapeutic options for dry eye disease.

#### 2.3.4. Contact Lenses and the Ocular Surface (Lipidure)

Wearing contact lenses can pose significant risks to the ocular surface, primarily due to reduced tear film stability and the potential for desiccation, which can lead to discomfort, irritation, and even damage to the corneal epithelium. Extended lens wear can disrupt the natural balance of the tear film, exacerbating symptoms of dry eye disease (DED) and increasing inflammation. The mechanical friction of the lenses against the eye, combined with limited oxygen permeability and the disruption of normal tear exchange, contributes to an environment that can compromise ocular health [56]. To address these concerns, effective lubricants are critical in maintaining ocular surface health and comfort during contact lens use. One promising lubricant is 2-methacryloyloxy ethyl phosphorylcholine (MPC; aka Lipidure), a synthetic polymer designed to mimic the properties of natural tears and provide lasting hydration and protection to the ocular surface [57].

In a preclinical study, we evaluated Lipidure for its effectiveness when used as an additive in artificial tears [58]. The research demonstrated that Lipidure significantly preserves corneal epithelial cell viability in cultures exposed to desiccating conditions, outperforming conventional lubricants such as trehalose and hyaluronic acid. The study involved in vitro assays utilizing rabbit corneal cells and ex vivo evaluations assessing the integrity of rabbit corneas subjected to desiccation. The results indicated that Lipidure effectively reduced cell death and maintained epithelial integrity, as evidenced by assays measuring cell viability and morphology. This protective effect was associated with a reduction in matrix metalloproteinase (MMP) activity, suggesting that Lipidure not only provides lubrication but also plays a crucial role in minimizing inflammation and oxidative stress on the ocular surface.

In conjunction with the preclinical findings, clinical evidence from Gagliano et al. [59] further substantiated the efficacy of Lipidure for contact lens wearers. In this randomized, controlled trial involving 30 participants, we compared the effects of artificial tears containing Lipidure and hypromellose (HPMC) to another popular formulation, Nextal (also HPMC-based). Over 21 days, patients were instructed to administer the drops three times daily, with outcomes measured using a range of parameters, including the visual analogue scale (VAS) for comfort, symptom assessments via the SANDE score, and non-invasive first break-up time (NIF-BUT). The results showed statistically significant improvements in both comfort and overall dryness relief in patients using Lipidure, mirroring the outcomes seen in the control group. Importantly, there were no significant differences between the two groups regarding adverse events, indicating that Lipidure is both safe and effective for patient use. The study concluded that Lipidure combined with HPMC demonstrates non-inferiority to Nextal, making it a valuable treatment option for individuals suffering from DED, particularly those reliant on contact lenses. Additionally, the research underscores the potential of Lipidure to not only serve as a lubricant but also enhance the resilience of the ocular surface, addressing common challenges faced by contact lens wearers. The dual role of Lipidure as a moisturizer and protective agent exemplifies its therapeutic promise in improving patient comfort and ocular surface health.

### 2.4. Meldonium

Scarring following eye surgery—particularly glaucoma filtration surgery (GFS)—poses a major challenge to surgical success, as conjunctival fibrosis can impair aqueous humor drainage and IOP [60]. Traditionally, mitomycin C (MMC) has been used intraoperatively to mitigate scarring by suppressing fibroblast proliferation. However, its clinical utility is limited by toxicity concerns and transient efficacy, which may compromise surgical outcomes [61]. Recent studies suggest that transepithelial meldonium (MID) eye drops may serve as a safer and more effective therapeutic alternative. Our research demonstrated that meldonium inhibits cell motility—a mechanism that could prevent pathological scarring and conjunctival overgrowth in conditions such as pterygium and pinguecula [62].

In normotensive rats, we first established meldonium’s additional benefit in reducing IOP through topical nanomicellar formulations [62]. The study showed a significant, dose-dependent IOP reduction of 25–32% at 1% and 2% concentrations, respectively, highlighting its potential as an IOP-lowering agent.

Further in vitro investigations revealed meldonium’s ability to markedly reduce cell motility in human trabecular meshwork (HTM) cells and scleral fibroblasts. Scratch wound healing assays demonstrated that meldonium effectively inhibited HTM cell migration—a key process in post-surgical scar formation. This anti-migratory effect was associated with cytoskeletal reorganization, evidenced by increased vinculin expression, which stabilizes focal adhesions and regulates cell movement.

The nanomicellar eye drop formulation may enhance meldonium’s ocular penetration, potentially enabling sustained therapeutic effects with minimal toxicity—a significant advantage over conventional antifibrotic agents. In fact, unlike MMC—which must be applied intraoperatively due to its toxicity—meldonium’s transepithelial eye drops enable non-invasive, post-surgical scar management. Given its favorable safety profile and dual mechanisms of action (IOP reduction and anti-fibrotic effects), meldonium emerges as a promising candidate for preventing post-surgical scarring and managing conjunctival overgrowth in pterygium/pinguecula. This innovative approach could transform post-operative care and improve outcomes in ocular surgery.

### 2.5. Keratoconus

Keratoconus arises from stromal thinning due to disrupted collagen architecture and aberrant keratocyte activity, exacerbated by oxidative stress. Progressive ectasia leads to visual impairment, often necessitating surgical intervention in advanced stages [63]. Corneal crosslinking (CXL) is a well-established surgical technique designed to strengthen the corneal tissue, primarily used in the treatment of keratoconus and post-refractive surgery ectasia. The procedure involves the application of riboflavin (vitamin B2) to the cornea, followed by exposure to ultraviolet A (UV-A) light. The riboflavin, once activated by the UV-A light, forms covalent bonds between collagen fibers in the corneal stroma, thereby inducing biomechanical stiffening and improving corneal stability [63]. A critical factor influencing the success of CXL is the efficient and homogeneous diffusion of riboflavin into the corneal stroma. Insufficient riboflavin penetration can lead to inadequate crosslinking, potentially resulting in failure to halt or improve corneal ectasia. Traditionally, the riboflavin solution is delivered after performing a debridement of the corneal epithelium, exposing the underlying stroma directly to the treatment. However, this method can be painful and involves a sterile surgical environment, adding complexities to the procedure [63].

The need for a less invasive and painful method of riboflavin permeation is underscored by two consecutive preclinical studies focusing on transepithelial corneal crosslinking techniques. Labate and colleagues [64] examined a novel transepithelial corneal crosslinking method that employed a nanotechnology-based riboflavin formulation that we designed to enhance riboflavin penetration through the intact epithelium. The study demonstrated that this approach resulted in a significant increase in the mechanical strength of the cornea, with findings indicating that the Young’s modulus after treatment with the nanoplatform was significantly greater than that of untreated controls. This method can minimize the pain and discomfort associated with epithelial removal, thereby improving the patient experience and reducing the recovery time. Next, we investigated the intrastromal riboflavin concentration in a transepithelial setting, showing that the use of this novel 0.1% riboflavin solution improved riboflavin diffusion across the intact epithelium compared to traditional methods [65]. These findings revealed that although the mean stromal concentration of riboflavin was lower in the nanotechnology group than in the deepithelialized control group, the higher bioavailability of riboflavin with less invasive methods could effectively facilitate crosslinking while maintaining patient comfort. The reduced need for a sterile surgical environment when utilizing the transepithelial approach enhances its practicality, allowing for broader clinical application and potentially increasing accessibility for patients. This evolution in CXL techniques not only promises to make the procedure less painful but also emphasizes the importance of advancing methods to optimize riboflavin diffusion for more effective and safer corneal crosslinking outcomes. Overall, the integration of transepithelial methods in corneal crosslinking represents a significant step forward in enhancing patient comfort and operational efficiency, showcasing the potential of innovative approaches in improving ocular surgical techniques.

Building on these findings, Lombardo et al. pioneered a theranostic approach to corneal cross-linking (CXL) for keratoconus, combining real-time diagnostic imaging with tailored therapeutic protocols. This innovation represents a major advance in personalized ophthalmology, optimizing treatment efficacy and safety outcomes [66]. Two consecutive clinical studies addressed the development and validation of theranostic imaging biomarkers that facilitate a real-time assessment of the riboflavin concentration and treatment efficacy during CXL procedures.

The first study [66] emphasizes the application of a theranostic device that allows for the simultaneous measurement of the corneal riboflavin concentration and a theranostic score, which predicts the biomechanical strengthening of the cornea post-treatment. Using a preclinical model, the researchers found a significant correlation between the theranostic score and the increase in mean corneal stiffness, highlighting the importance of ensuring sufficient riboflavin diffusion prior to UV-A irradiation for effective cross-linking.

The second study [67] builds on these findings through a randomized clinical trial involving fifty patients with progressive keratoconus. Participants were assigned to either epithelium-off or epithelium-on CXL protocols, utilizing the same theranostic device for controlled UV-A exposure while monitoring the riboflavin concentration. The trial’s results showcased the predictive power of the theranostic imaging biomarkers, achieving an impressive accuracy of 91% and a positive predictive value of 95% in forecasting a flattening of the maximum keratometry (K_max_) after one year. The median reduction of K_max_ by −1.3 diopters, along with significant improvements in uncorrected and corrected distance visual acuity, further validated the efficacy of this integrated approach.

The integration of theranostics into CXL procedures not only enhances the precision of treatment by tailoring interventions to the individual corneal profile but also streamlines the CXL process, potentially offering better outcomes with fewer complications. The findings underscore the critical role of the riboflavin concentration and its effect on cross-linking efficacy, reaffirming the theranostic approach as a promising paradigm in the management of keratoconus. By allowing for real-time adjustments based on immediate feedback from the theranostic parameters, clinicians can optimize treatment protocols, leading to more predictable and successful results in keratoconus therapy.

### 2.6. Eye Pain (Gabapentin)

Eye pain, particularly in moderate to severe DED, represents a significant clinical challenge that substantially impairs patients’ quality of life, making routine activities difficult due to persistent discomfort. The pathophysiology of ocular pain involves multiple mechanisms, including both nociceptive and neuropathic components, with the latter arising from nervous system dysfunction that may occur even in the absence of visible tissue damage [68]. Conventional therapies, primarily limited to palliative lubrication, often fail to address these underlying pain mechanisms, highlighting the need for more targeted therapeutic approaches.

Gabapentin (GBP), an FDA-approved neuropathic pain medication [69], emerges as a promising candidate for managing DED-related ocular pain. Unlike topical anesthetics that suppress corneal sensitivity and may compromise reflex tearing, GBP provides analgesia while preserving tear film homeostasis. Preclinical studies demonstrate that GBP exerts its effects by binding to the α2δ subunit of voltage-gated calcium channels, thereby reducing excitatory neurotransmitter release (particularly glutamate) while enhancing GABAergic activity [70]. This dual mechanism not only modulates pain perception but also helps control neurogenic inflammation at the ocular surface.

Our research has further elucidated GBP’s therapeutic potential in DED management through several key findings. Topical GBP application stimulates tear secretion by upregulating acetylcholine and norepinephrine signaling in lacrimal gland pathways [71]. Furthermore, it enhances aquaporin-5 (AQP5) expression in lacrimal tissue, which is crucial for maintaining tear film stability [71]. Additionally, GBP exerts anti-inflammatory effects through the suppression of pro-inflammatory cytokines and mediators [72]. These multifaceted actions position GBP as a comprehensive therapeutic agent capable of addressing both the symptomatic discomfort and underlying pathophysiology of DED.

The neuropathic component of DED-related pain, often refractory to conventional treatments [68], underscores the importance of developing targeted therapies like GBP. Its topical formulation represents a significant advancement in ocular pain management, particularly for patients with chronic DED or those unresponsive to standard therapies. This highlights the need for continued investigation into GBP’s mechanisms and the development of personalized treatment paradigms that account for the complex etiology of ocular pain.

In conclusion, topical gabapentin offers a novel therapeutic approach for DED, combining analgesic efficacy with tear film restoration and anti-inflammatory action. This comprehensive mechanism not only provides immediate symptom relief but also contributes to long-term ocular surface rehabilitation, representing a significant improvement over current palliative strategies for debilitating eye pain.

### 2.7. Ocular Surface Microbiota

The ocular surface microbiota (OSM) plays an essential role in maintaining ocular surface homeostasis, contributing to eye health by modulating immune responses, protecting against pathogens, and preserving the stability of the tear film. This diverse community of microorganisms, primarily consisting of bacteria, also includes fungi and viruses and interacts closely with the host’s immune system to enhance ocular health [73]. The tear film itself is rich in antimicrobial molecules, such as lysozyme and lactoferrin, which help regulate microbial populations and maintain a balanced ocular microbiome [74]. Epithelial cells on the ocular surface produce immunomodulatory cytokines, further contributing to this protective environment and acting as a physical barrier against pathogenic microbial invasion. For instance, certain commensal bacteria, like *Corynebacterium mastitidis*, stimulate the production of interleukin-17 (IL-17) by γδ T cells, promoting the release of antimicrobial proteins and bolstering the innate immune response without triggering excessive inflammation [74]. However, disturbances in this microbiota balance, known as dysbiosis, can lead to ocular surface pathologies such as DED, blepharitis, and conjunctivitis. Dysbiosis can result from factors such as antibiotic use, environmental changes, or systemic conditions, which favor pathogenic overgrowth, leading to inflammation and destabilization of the tear film [73].

Given the significant role of the OSM in ocular health, there is growing interest in therapeutic strategies aimed at restoring this delicate balance. One promising approach that I have most recently proposed involves integrating exogenous bacteria to enhance the ocular surface microbiota [74]. I suggested the use of a novel bioprinting technology to deliver beneficial bacterial strains in a solid form, overcoming the rapid clearance typically observed with conventional liquid bacterial suspensions. This bioprinted strategy not only could improve bacterial retention on the ocular surface but also should facilitate controlled release over time, thereby increasing the likelihood of successful colonization by beneficial microbes [74]. By effectively re-establishing a healthy microbial community, this approach could reduce the risk of infection, support tear film stability, and promote healing in chronic ocular surface conditions. The introduction of specific beneficial strains could outcompete harmful bacteria, produce antimicrobial compounds, and stimulate local immune responses, ultimately helping to prevent recurrent infections and maintain overall ocular health. Future studies will be essential to validate the safety and efficacy of this innovative therapeutic strategy, paving the way for more effective management of ocular surface diseases linked to microbiota dysbiosis.

### 2.8. Antibiotic/Steroid Eye Drops

However, when pathogenic bacteria invade the ocular surface, they can cause infections that require antibiotic treatment. Since these infections are invariably accompanied by inflammation, the concomitant use of a steroid may help resolve the pathology more rapidly. To test this hypothesis, we evaluated the enhanced therapeutic effects of combining the antibiotic ofloxacin with the anti-inflammatory dexamethasone in treating acute bacterial conjunctivitis, using a well-established rabbit model infected with *Staphylococcus aureus* or *Pseudomonas aeruginosa* [75]. The rabbit model was selected due to its anatomical and physiological similarities to the human eye, particularly in the conjunctival structure and immune response, making it a robust preclinical system for assessing ocular treatments. Our findings revealed that while ofloxacin alone effectively reduced the bacterial load and inflammatory cytokines (IL-6 and TGF-β) in tears, the addition of dexamethasone significantly accelerated clinical resolution, demonstrating a superior anti-inflammatory and antimicrobial synergy. These results underscore the potential of corticosteroid–antibiotic combinations to enhance treatment efficacy by simultaneously targeting infection and inflammation. Although preclinical, the strong translational relevance of this model, which closely mirrors human disease, provides compelling support for further clinical investigation into such combination therapies for bacterial conjunctivitis.

## 3. Glaucoma

Glaucoma, particularly primary open-angle glaucoma (POAG), is one of the leading causes of irreversible blindness worldwide, characterized by the progressive degeneration of retinal ganglion cells (RGCs) and subsequent visual field loss [76]. POAG is often asymptomatic in its early stages, leading to a significant problem of late diagnosis; by the time patients present with overt symptoms, substantial visual impairment may already have occurred, complicating management efforts.

### 3.1. The Role of the Trabecular Meshwork

The dominant role of IOP in the genesis and management of POAG cannot be overstated. Elevated IOP, a major risk factor for glaucomatous optic neuropathy, arises primarily from the impaired outflow of AH through the TM, the eye’s primary drainage pathway. The TM regulates AH outflow via its extracellular matrix (ECM), where glycosaminoglycans (GAGs)—particularly HA and sulfated GAGs like chondroitin sulfate—play a critical role in modulating outflow resistance. We have demonstrated that in POAG and aging eyes, the TM undergoes pathological remodeling: the HA content decreases significantly, while sulfated GAGs (e.g., chondroitin and dermatan sulfate) accumulate, correlating with increased ECM electron density and fibrous deposits. These changes stiffen the TM, narrowing Schlemm’s canal and exacerbating outflow resistance [77]. Notably, glaucomatous eyes exhibited a 3-fold reduction in HA and a 2.8-fold increase in chondroitin sulfate compared to young controls, directly linking GAG dysregulation to IOP elevation. This mechanical stress, compounded by AH retention, perpetuates retinal ganglion cell damage, underscoring why TM-targeted therapies—such as HA restoration or sulfated GAG inhibition—are emerging as complementary strategies to conventional IOP-lowering treatments.

### 3.2. Intraocular Pressure (IOP) and Corneal Temperature

The IOP remains the only modifiable risk factor in glaucoma, establishing it as a critical target for both understanding and managing this sight-threatening condition [78]. Recognizing this fundamental relationship, we investigated the interplay between the corneal temperature and IOP—two key elements in glaucoma pathophysiology [79]. Our findings demonstrate that corneal temperature variations significantly affect IOP levels, suggesting that both thermal conditions and oxidative stress may influence aqueous humor dynamics. Specifically, our research identified that therapeutic interventions like cooling mask application and eyelid closure induce measurable changes in the corneal temperature that correlate with IOP fluctuations. Given the central role of IOP control in glaucoma management, these temperature-dependent effects could pave the way for novel non-pharmacological approaches to IOP regulation. The potential incorporation of temperature-modulating techniques, particularly cooling protocols, may offer valuable adjunctive therapy options—especially for managing IOP spikes that often challenge conventional treatment strategies. This study advances our comprehension of glaucoma pathophysiology while introducing innovative possibilities for therapeutic intervention. By elucidating the temperature–IOP relationship, our work contributes to the development of complementary strategies aimed at IOP stabilization and optic nerve protection, potentially enhancing overall glaucoma management outcomes.

### 3.3. Neuroprotection

**Figure 2:**
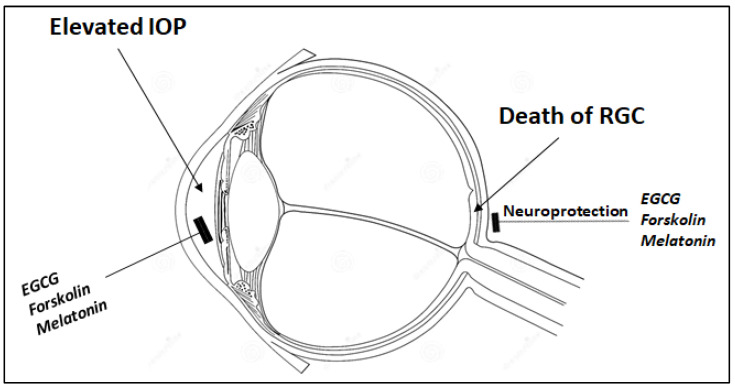
Glaucoma IOP and neuroprotection. The major risk factor for POAG is the elevated IOP, which in turn leads to the apoptotic death of RGCs. Recent studies have identified natural molecules, such as EGCG, forskolin, and melatonin, capable of both reducing IOP and protecting RGCs from apoptosis. All three molecules demonstrate potent antioxidant activity along with direct and indirect neuroprotective effects.

The current standard treatment for POAG, while effective at lowering IOP to prevent optic nerve damage, fails to address the underlying neurodegenerative processes causing RGC loss. This critical gap underscores the urgent need for complementary neuroprotective strategies targeting key pathological mechanisms—including oxidative stress, neuroinflammation, and glutamate excitotoxicity—that drive RGC degeneration [80]. Years before, we had proposed that integrating such neuroprotective approaches with conventional IOP-lowering therapies could potentially slow disease progression and preserve visual function [81], particularly given that approximately one-third of POAG patients exhibit normal or low IOP—clear evidence that mechanisms beyond pressure dysregulation contribute to disease pathogenesis (Figure 2).

#### 3.3.1. Glaucoma and Alzheimer’s

Our findings further support this therapeutic approach by revealing striking parallels between POAG and Alzheimer’s disease (AD) pathophysiology [82]. The shared features—including amyloid-β peptide accumulation and chronic neuroinflammatory processes—not only explain RGC degeneration in both conditions but also suggest that neuroprotective therapies developed for AD may benefit glaucoma management. These insights advocate for a paradigm shift toward comprehensive treatment strategies that simultaneously target IOP regulation and the underlying neurodegeneration. By addressing both aspects of the disease pathology, this dual approach could improve outcomes for glaucoma patients while potentially mitigating the associated risks of cognitive decline [82].

#### 3.3.2. Epigallocatechin Gallate (EGCG)

Building upon this foundation, we investigated the neuroprotective potential of epigallocatechin gallate (EGCG), a bioactive flavonoid abundant in green tea, for its therapeutic effects on RGC degeneration in glaucoma. In collaborative preclinical studies with Prof. N. Osborne’s group, we demonstrated EGCG’s capacity to protect RGCs against ischemia/reperfusion injury—a pathological condition closely associated with elevated IOP. Our experimental model of artificially induced ischemia in rats produced significant retinal dysfunction, characterized by reduced electroretinogram (ERG) amplitudes and elevated apoptotic markers, including caspase activation and the increased expression of pro-apoptotic proteins [83,84].

Notably, pretreatment with EGCG effectively preserved retinal functions, as evidenced by maintained ERG amplitudes, while simultaneously suppressing apoptotic markers [83]. These findings demonstrate EGCG’s ability to counteract cell death pathways triggered by oxidative stress and excitotoxicity. Mechanistic studies revealed that EGCG’s neuroprotective effects stem from its potent antioxidant activity, which scavenges reactive oxygen species (ROS) and reduces oxidative damage in retinal tissues. Further investigation of the NF-κB signaling pathway showed that EGCG treatment inhibits the phosphorylation of key inflammatory mediators, thereby attenuating optic nerve injury in acute glaucoma models [85]. This anti-inflammatory action is particularly significant given the established role of neuroinflammation in glaucoma progression.

Moreover, our investigation extended to oral EGCG administration, which demonstrated comparable neuroprotective effects against ischemic retinal damage [84]. This study further revealed EGCG’s capacity to regulate gene expression networks governing cell survival and apoptosis, expanding its mechanistic profile beyond antioxidant activity. These findings hold particular relevance for glaucoma management, where RGC degeneration often progresses despite adequate IOP control.

The therapeutic implications are twofold: EGCG may complement conventional care by both contributing to IOP reduction [86] and providing direct neuroprotection. Our preclinical evidence thus positions EGCG as a promising candidate for adjunctive therapy in glaucoma—one that could address both the symptomatic (IOP elevation) and neurodegenerative aspects of the disease. This dual-action potential represents a significant advancement over current treatment paradigms focused solely on pressure management.

Translating these mechanistic insights to clinical practice, we evaluated EGCG’s short-term effects in a clinical study involving ocular hypertension and primary open-angle glaucoma patients. Following oral EGCG supplementation, participants demonstrated significant improvements in pattern electroretinogram (PERG) amplitudes—a key indicator of enhanced inner retinal function, particularly in early-to-moderate disease stages [87]. While long-term neuroprotective outcomes require further investigation, these immediate functional improvements provide clinically relevant evidence that dietary bioactive compounds like EGCG may augment standard therapies through two complementary mechanisms: (1) direct modulation of neuroprotective pathways and (2) functional enhancement of retinal signaling. These findings bridge an important gap between experimental models and clinical applications, suggesting EGCG’s potential as a nutritional adjunct in comprehensive glaucoma management strategies.

More recent research has made significant progress in understanding and corroborating the neuroprotective properties of EGCG in the context of glaucoma, further supporting its potential as a therapeutic agent. In a mouse model of chronic glaucoma, EGCG administration significantly protected retinal ganglion cells (RGCs) from degeneration induced by elevated intraocular pressure (IOP) through a microbead injection method. The study revealed that mice receiving EGCG in their drinking water displayed a notably higher density of RGCs compared to those subjected to elevated IOP without EGCG treatment, highlighting a clear neuroprotective effect of this compound in preventing RGC loss [88]. This neuroprotection was attributed to various mechanisms, including the antioxidant properties of EGCG which combat oxidative stress—a contributing factor in neurodegeneration within the retina.

Additionally, EGCG has been shown to enhance autophagic activity in other ocular cells, which is crucial for cellular health and function. EGCG was shown to effectively counter the inhibitory effects of transforming growth factor beta-1 (TGF-β1), a cytokine associated with fibrosis and abnormal cell transformation in human Tenon’s fibroblasts. By promoting the autophagic process, EGCG demonstrated its potential as an antifibrotic agent, thereby providing a dual benefit not only for neuroprotection but also in reducing scarring complications following glaucoma surgery [89].

In other in vitro investigations, EGCG’s efficacy in protecting trabecular meshwork (TM) cells from endoplasmic reticulum (ER) stress has also been highlighted. A pre-treatment with EGCG significantly improved the viability of TM cells under ER stress conditions, alleviating the pathological mechanisms that can lead to increased IOP in primary open-angle glaucoma (POAG) patients [90].

Collectively, these findings underscore the multifaceted neuroprotective effects of EGCG in glaucoma, addressing the critical need for therapies that not only focus on IOP reduction but also target the underlying neurodegenerative processes associated with RGC loss. The compound not only aids in preserving RGC health but also mitigates fibrotic transformations in fibroblasts and improves the functioning of trabecular meshwork cells, thereby addressing various pathogenic mechanisms associated with glaucoma. This approach emphasizes the promising role of EGCG in enhancing the neuroprotective landscape in glaucoma management, suggesting that integrating this natural compound into treatment regimens could complement traditional therapies aimed solely at lowering IOP [81].

##### Antibacterial Synergy of EGCG: Implications for Ocular Infections

Beyond its neuroprotective and antioxidant roles, EGCG exhibits potent antibacterial properties, particularly against *Staphylococcus* spp., a common pathogen in ocular infections (e.g., bacterial keratitis, endophthalmitis). We have demonstrated that EGCG at subinhibitory concentrations (15–50 µg/mL) reverses tetracycline resistance in staphylococci by inhibiting the Tet(K) efflux pump, leading to increased intracellular tetracycline accumulation [91]. This synergy was observed in both resistant and susceptible strains, with a 256-fold reduction in tetracycline MICs for resistant *S. epidermidis*. Spectrofluorometric assays confirmed EGCG’s mechanism: blocking efflux pumps (including Tet(B)), thereby enhancing drug retention. These findings suggest EGCG’s potential as an adjuvant in ocular antibiotic therapy, particularly for infections involving multidrug-resistant staphylococci. While this study focused on systemic applications, its implications extend to topical ophthalmic formulations, where EGCG could augment conventional antibiotics while mitigating resistance—a critical consideration in perioperative or chronic ocular surface infections.

##### Alternative Administration Routes

In light of the potential therapeutic roles of epigallocatechin gallate (EGCG) in neuroprotection against glaucoma, exploring alternative administration routes could significantly enhance its bioavailability and effectiveness. Recent innovations in drug delivery systems have illuminated promising strategies for EGCG administration beyond traditional oral methods. For instance, a study by Pan WY et al. [92] developed a novel intranasal delivery system utilizing platelet extracellular vesicles (pEVs) as carriers for EGCG. This approach exploits the nose-to-brain pathway, allowing for direct delivery to the retinal tissues while circumventing biological barriers associated with traditional systemic administration. The intranasal administration of EGCG-pEVs demonstrated superior therapeutic efficacy, including reduced intraocular pressure and anti-inflammatory effects in an animal model of glaucoma, suggesting a significant advancement in drug distribution and effectiveness in managing the disease. Moreover, Fangueiro et al. [93] explored the encapsulation of EGCG within lipid nanoparticles (LNs), a strategy designed to protect the compound from oxidation and enhance its stability for ocular applications. These LNs were engineered to optimize ocular retention and mucoadhesion through the use of cationic lipids, promoting prolonged drug contact with the ocular surface, which is crucial for effective treatment outcomes. The study reported that these lipid nanoparticles complied with ocular administration parameters, indicating their potential for delivering EGCG directly to the affected retinal tissues in various ocular diseases, including glaucoma.

Together, these alternative administration strategies not only improve the pharmacokinetics of EGCG but also hold promise for enhancing its therapeutic efficacy as a neuroprotective agent in glaucoma management. By integrating such innovative delivery systems with existing treatment protocols, clinicians could provide a more robust approach to preserving retinal health and preventing progression in patients afflicted with this debilitating condition.

#### 3.3.3. Forskolin

Forskolin, a natural compound extracted from *Coleus forskohlii*, has emerged as a multifaceted therapeutic agent in glaucoma, uniquely capable of addressing both the biomechanical and neurodegenerative components of the disease. By directly activating adenylate cyclase, forskolin elevates intracellular cAMP levels, triggering a cascade of biologically significant effects. Its IOP-lowering action stems from the dual modulation of aqueous humor dynamics: not only does it suppress aqueous production by inhibiting ion transport in the ciliary epithelium, but it also enhances reabsorption from the posterior chamber into the ciliary stroma through cAMP-mediated signaling across the bilayer ciliary epithelium. These actions complement its well-documented effect of improving the trabecular meshwork outflow facility [94,95,96].

Beyond aqueous humor regulation, forskolin’s neuroprotective potential is equally compelling. The sustained cAMP elevation activates critical survival pathways in RGCs, most notably through the potentiation of Brain-Derived Neurotrophic Factor (BDNF) signaling. By facilitating the translocation of TrkB receptors to RGC membranes and downstream activation of CREB and PI3K/Akt pathways [94], forskolin creates a robust pro-survival environment that counters glaucomatous neurodegeneration [97,98]. This dual capacity to simultaneously normalize IOP while reinforcing RGC resilience positions forskolin as a uniquely comprehensive therapeutic candidate, potentially bridging the gap between current pressure-lowering strategies and the unmet need for effective neuroprotection in glaucoma management.

##### Kronek^®^

In a pivotal preclinical study, we demonstrated that forskolin indeed enhanced neuroprotection in a rat model of experimental hypertensive glaucoma [99]. In the experiment, ocular hypertension was induced in rats by injecting 2% methylcellulose into the anterior chamber, blocking aqueous humor outflow and raising IOP. Forskolin (1 mg/kg) was administered orally to half the rats one week before IOP elevation. Results showed that forskolin significantly reduced IOP and retinal damage. TUNEL assays revealed less apoptosis in the RGCs of forskolin-treated rats, with reduced caspase-3 and inducible nitric oxide synthase (iNOS) levels. Therefore, forskolin’s dual action—lowering the IOP and promoting RGC survival—confirms its potential as a neuroprotective agent in glaucoma.

In clinical practice, an oral forskolin–rutin combination (Kronek^®^) therapy has demonstrated significant efficacy in reducing intraocular pressure (IOP), particularly in patients with poorly controlled glaucoma despite maximal medical therapy. Clinical studies report a mean IOP reduction of 10–15%, with more pronounced effects (15% decrease) observed in patients with elevated baseline IOP (≥21 mmHg) compared to those with lower pressures (9% decrease) [100]. This supplemental IOP-lowering effect is clinically valuable for high-risk patients awaiting surgical intervention.

Beyond pressure control, this combination therapy shows promising neuroprotective potential. A randomized clinical trial involving 45 primary open-angle glaucoma patients revealed that adjunctive forskolin–rutin treatment not only augmented the IOP reduction but also significantly improved Pattern Electroretinogram (PERG) amplitudes—a key indicator of retinal ganglion cell (RGC) function [101]. Mechanistically, while rutin contributes primarily through vascular effects (enhancing ocular perfusion and mitigating ischemic damage), forskolin exerts dual therapeutic effects: (1) cAMP/PKA-mediated IOP reduction via aqueous humor dynamics modulation, and (2) direct neuroprotection through anti-apoptotic pathways in RGCs [100,101].

These findings position the forskolin–rutin combination as a unique adjunctive therapy that addresses both the biomechanical and neurodegenerative aspects of glaucoma. By concurrently lowering IOP and preserving RGC functions, this approach may be particularly beneficial for patients with progressive disease despite conventional treatment, offering a valuable bridge to surgical intervention while potentially slowing disease progression.

In conclusion, the combined use of EGCG and forskolin might represent a novel, mechanistically complementary approach to glaucoma therapy that simultaneously addresses both the neurodegenerative and biomechanical aspects of the disease. EGCG exerts comprehensive neuroprotection through three primary pathways: (1) potent antioxidant activity that neutralizes reactive oxygen species, (2) suppression of neuroinflammatory cascades, and (3) direct inhibition of retinal ganglion cell (RGC) apoptosis. These effects collectively mitigate the oxidative stress and inflammatory damage that drive glaucomatous optic neuropathy. Forskolin complements these actions through cAMP-mediated mechanisms that both reduce intraocular pressure (via enhanced trabecular meshwork outflow and uveoscleral drainage) and promote RGC survival (through BDNF/TrkB pathway activation). The synergistic potential of these compounds lies in their ability to target distinct yet interconnected pathological processes: while EGCG protects against molecular-level damage, forskolin addresses both the hydrodynamic dysfunction (IOP elevation) and provides additional neurotrophic support. This multi-target strategy may offer superior clinical outcomes compared to monotherapies, particularly for patients with progressive disease despite IOP control [102]. Future investigations should prioritize the following: (1) development of optimized delivery systems to enhance ocular bioavailability, (2) determination of ideal dosing ratios for maximal synergy, and (3) rigorous clinical validation through randomized controlled trials assessing both structural and functional endpoints in glaucoma patients.

##### Gangliolife^®^ and Gangliomix^®^: Novel Nutraceutical Approaches to Glaucoma Neuroprotection

As research on the forskolin–EGCG combination advanced, parallel investigations were developing innovative neuroprotective formulations for glaucoma. This work led us to two strategically designed nutraceuticals—Gangliolife^®^ and Gangliomix^®^—that combine multiple bioactive compounds targeting different aspects of glaucoma pathophysiology. Central to both formulations is homotaurine, a naturally occurring aminosulfonate compound that plays a pivotal neuroprotective role by modulating neuronal excitability, reducing excitotoxic damage, and inhibiting pathological protein aggregation.

These advanced formulations integrate homotaurine’s unique properties with other carefully selected components: forskolin’s dual ability to modulate IOP and protect retinal ganglion cells (RGCs), along with essential B vitamins that support neuronal metabolism and combat oxidative stress. In Gangliolife^®^, this combination is further enhanced by the addition of L-carnosine, a potent antioxidant that specifically targets oxidative stress—a key contributor to RGC degeneration.

The efficacy of this multi-target approach was demonstrated in collaborative research with Russo et al. [103], where Gangliolife^®^ showed significant neuroprotective effects in an acute angle-closure glaucoma model. The formulation’s synergistic action resulted in markedly reduced RGC apoptosis, as evidenced by decreased caspase-3 activation and fewer TUNEL-positive cells, confirming its ability to enhance neuronal survival under pathological conditions.

This strategic combination of compounds represents an evolution in nutraceutical therapy for glaucoma, moving beyond single-agent approaches to address the complex, multifactorial nature of glaucomatous neurodegeneration while maintaining an excellent safety profile for chronic use.

The clinical potential of Gangliolife^®^ lies in its unique capacity to simultaneously address both intraocular pressure (IOP) regulation and retinal ganglion cell (RGC) neuroprotection—two critical aspects of glaucoma management. This dual-action profile was clinically validated in a 12-month pilot study by Mutolo et al. [104] involving 22 primary open-angle glaucoma (POAG) patients (44 eyes) receiving standard IOP-lowering therapy. The Gangliolife^®^-treated group demonstrated statistically significant improvements across multiple parameters: a sustained 1.9 mmHg reduction in IOP, coupled with progressive enhancements in pattern electroretinogram (PERG) amplitudes and foveal sensitivity at 6, 9, and 12-month follow-ups. These functional improvements, absent in the control group, suggest the successful ocular bioavailability of Gangliolife^®^’s active components and their ability to exert both biomechanical and neuroprotective effects. The temporal pattern of improvement—with retinal functional parameters continuing to enhance throughout the study period—points to cumulative neuroprotective benefits that complement conventional IOP-lowering approaches. These findings position Gangliolife^®^ as a promising adjunctive therapy capable of addressing the multifactorial nature of glaucoma progression.

Building upon Gangliolife’s neuroprotective foundation, Gangliomix^®^ was developed as an enhanced formulation incorporating additional bioactive components—spearmint extract and optimized B-vitamin complexes—to create a more comprehensive therapeutic approach. Its efficacy was systematically evaluated using two complementary glaucoma models, each revealing distinct protective mechanisms.

In the optic nerve crush (ONC) model [105], which replicates the acute axonal injury characteristic of glaucoma, Gangliomix^®^ demonstrated robust preservation of retinal ganglion cell (RGC) functions. Treatment not only maintained photopic negative response (PhNR) amplitudes, reflecting intact RGC signaling, but also significantly downregulated apoptotic markers (GFAP and iNOS). The formulation’s ability to favorably modulate the Bax/Bcl-2 ratio while suppressing caspase-3 activation indicates potent interference with both intrinsic and extrinsic apoptotic pathways, suggesting a dual mechanism of action combining anti-inflammatory effects with direct cytoprotection.

The hypertensive glaucoma model [106], involving chronic intraocular pressure elevation via anterior chamber methylcellulose injection, provided further evidence of the therapeutic potential of Gangliomix^®^. Under sustained ocular hypertension, the formulation preserved key visual function parameters while mitigating neuroinflammatory responses mediated by activated glial cells. This multimodal efficacy arises from strategic synergy between its components: forskolin’s dual IOP-modulating and neurotrophic actions, homotaurine’s calcium channel modulation and anti-excitotoxic effects, coupled with the combined antioxidant capacity of spearmint polyphenols and B vitamins’ support of mitochondrial function. By simultaneously addressing both the mechanical stress of elevated IOP and its downstream neurodegenerative consequences, Gangliomix^®^ emerges as a promising disease-modifying strategy for comprehensive glaucoma management.

In summary, both Gangliolife^®^ and Gangliomix^®^ represent promising advancements in the fight against glaucoma. Their ability to target multiple pathways involved in RGC survival and disease progression holds significant potential for improving clinical outcomes in patients with glaucoma. By leveraging a combination of natural compounds, these formulations could offer enhanced neuroprotection and serve as critical adjuncts to current IOP-reducing therapies. Further clinical validation is indispensable for establishing their place in standard glaucoma management protocols, ultimately improving the quality of life for those at risk of vision loss.

##### Kronek^®^ in Nd:YAG Laser Iridotomy

Moreover, another clinical study, although on a different type of patients, further underscored the versatility of forskolin in glaucoma management by demonstrating its efficacy in preventing acute intraocular pressure (IOP) spikes following Nd:YAG laser iridotomy, a procedure commonly used to treat patients at risk of angle-closure glaucoma [107]. In this double-blind, placebo-controlled study, patients pretreated with an oral combination of forskolin (15 mg) and rutin (200 mg) exhibited no significant IOP elevation post-iridotomy, whereas placebo-treated patients experienced a pronounced spike (average 3 mmHg) that persisted for days. The forskolin–rutin association not only stabilized IOP but also mitigated the risk of optic nerve damage associated with such pressure fluctuations. This aligns with forskolin’s known mechanisms: by activating adenylate cyclase and increasing cAMP, it likely enhances aqueous humor outflow through trabecular meshwork relaxation while counteracting inflammation-induced outflow resistance. Rutin’s complementary effects—improving ocular blood flow and reducing oxidative stress—may further protect tissues from laser-induced trauma. These findings highlight a novel adjunctive application of forskolin, particularly in procedural settings where transient IOP elevation poses a clinical challenge. The study reinforces the broader potential of forskolin-based therapies, bridging its mechanistic benefits with practical, scenario-specific utility in glaucoma care.

##### Topical Forskolin Formulations

Finally, we also investigated topical forskolin formulations by developing in situ gelling eye drops for glaucoma treatment [108]. Three formulations—thermo-sensitive (Pluronic^®^), pH-sensitive (Carbopol^®^), and ion-sensitive (gellan gum)—were tested for gelation, stability, and drug release. The pH-sensitive gel showed the best performance, delivering forskolin effectively to the cornea and aqueous humor, comparable to aqueous suspensions, while thermo-sensitive gels released the drug more slowly. All formulations remained stable for six months. The results suggest that pH-sensitive in situ gels could enhance forskolin’s ocular bioavailability, offering a promising alternative to conventional eye drops with improved retention and sustained release. Further in vivo studies are needed to confirm tolerability and efficacy.

#### 3.3.4. Citicoline

Citicoline, also known as cytidine 5′-diphosphocholine, is a naturally occurring cytidine compound that serves as a precursor for the synthesis of phosphatidylcholine, a critical component of cellular membranes. Its neuroprotective properties have garnered attention in the context of glaucoma. Studies indicate that citicoline promotes neuronal survival and functionality through various mechanisms, including enhancing mitochondrial function, reducing oxidative stress, and modulating inflammatory pathways [109]. Research has demonstrated that citicoline administration can lead to significant improvements in visual function in patients with glaucoma. Clinical trials by Rossetti et al. [110,111] reported that citicoline supplementation resulted in improved quality of life, visual field sensitivity, and a reduction in visual field deterioration in glaucomatous patients. Furthermore, preclinical studies have shown that citicoline administration can significantly protect against RGC loss in experimental models of optic nerve lesions by enhancing axonal regeneration and preserving optic nerve integrity [112,113]. These findings suggest that citicoline may serve as a valuable adjunctive therapy for patients with glaucoma, targeting the neurodegenerative aspects of the disease while complementing traditional IOP-lowering treatments. Ongoing research into its long-term efficacy and optimal dosing regimens will be crucial for establishing citicoline’s role in comprehensive glaucoma management.

#### 3.3.5. Niacin

Based on these findings, we formulated a new neuroprotective food supplement combining citicoline and niacin. Niacin, also known as vitamin B3, has emerged as a potential neuroprotective agent in glaucoma treatment due to its diverse physiological roles, particularly in neural health. Research suggests that niacin may help mitigate oxidative stress and inflammation, both of which play a critical role in retinal ganglion cell (RGC) degeneration in glaucoma. Recent studies highlight niacin’s neuroprotective effects, particularly in trauma and ischemic conditions, which could be beneficial in glaucoma management. In a study by Ermutlu et al. [114], niacin demonstrated significant neuroprotective effects against spinal cord ischemia/reperfusion injury in rabbits, reducing oxidative stress markers and apoptosis while improving neurological outcomes. Similarly, Ozaydin et al. [115] found that niacin treatment following mild traumatic brain injury in rats decreased oxidative stress and inflammation while improving histopathological damage.

These findings suggest that niacin’s ability to combat oxidative stress and inflammation could also aid in protecting RGCs in glaucoma, potentially complementing traditional therapies that primarily target IOP. Its mechanisms also include promoting vascular health, enhancing blood flow to the optic nerve, and stimulating the release of neuroprotective factors. Studies have indicated that niacin can improve visual function and slow disease progression in glaucoma models, highlighting its possible benefits as an adjunctive therapy alongside traditional IOP-lowering treatments [116,117]. Furthermore, niacin’s potential to modulate lipid metabolism and reduce inflammation [117] positions it as a promising candidate for exploring additional strategies to protect RGCs and preserve vision in patients with glaucoma.

#### 3.3.6. Citicoline + Niacin

In order to evaluate the combined effects of niacin and citicoline on the protection of RGCs, we used a mouse model of hypertensive glaucoma induced by MCE injection in the anterior chamber of the eye [118]. Results indicated that the optimal combination of the active molecules preserved approximately 50% of RGCs from degeneration, with corresponding functional improvements evident in electroretinography recordings. While high-dose monotherapies of either compound nearly normalized photopic negative response (PhNR) amplitudes, the combined treatment—particularly low-dose citicoline with varying niacin doses—demonstrated superior protection of RGC density and function. The combination therapy’s efficacy appeared to be mediated by multiple complementary pathways. Oxidative stress markers (Nrf2, HO-1) were halved, while inflammatory mediators (pNF-κB, IL-6) showed significant suppression. Notably, we observed reduced caspase-3 activity and improved Bax/Bcl-2 ratios, indicating enhanced mitochondrial stability and attenuated apoptosis. These findings position the niacin–citicoline combination as a promising multi-target therapeutic strategy that simultaneously addresses oxidative damage, neuroinflammation, and apoptotic signaling in glaucomatous neurodegeneration.

Therefore, the combined use of niacin and citicoline demonstrates synergistic neuroprotection in hypertensive glaucoma by targeting complementary pathways critical for RGC survival. Niacin, as a precursor to NAD+, bolsters mitochondrial function and energy metabolism—key factors compromised in glaucoma patients. By maintaining NAD+ levels, it preserves ATP production and mitigates oxidative damage. Citicoline complements this action by stabilizing neuronal membranes through enhanced phospholipid synthesis, including cardiolipin, while also supplying choline for acetylcholine production to support neuroprotective cholinergic signaling.

Together, these compounds address the triad of glaucoma pathogeneses: oxidative stress, neuroinflammation, and apoptotic signaling. Their combination not only surpasses individual treatments in preserving RGC density and function but may enable lower effective doses, minimizing potential side effects. This multitarget strategy represents a promising dietary intervention to augment conventional glaucoma therapies, offering a novel approach to slow neurodegeneration and preserve vision.

#### 3.3.7. The Final Mix

Interestingly, and in line with previous suggestions [102], a novel nutraceutical formulation based on the aforementioned research has been developed and marketed under the trade name Epicolin^®^. It combines the neuroprotective agents forskolin, homotaurine, EGCG, and citicoline [119]. A recently published preclinical study explores the neuroprotective, antioxidant, and anti-inflammatory effects of this novel nutraceutical formulation. Using both in vitro and in vivo models, the researchers assessed Epicolin^®^’s effectiveness in protecting RGCs from degeneration. Results indicated that Epicolin^®^ significantly enhanced cell viability and neuroprotection in neuroblastoma (SH-SY5Y) cells challenged by neurotoxic agents, showing superior efficacy compared to other marketed formulations. In vivo studies conducted on DBA/2J glaucomatous mice demonstrated that Epicolin^®^ treatment improved RGC function as measured by PERG and reduced RGC death by enhancing BDNF levels while lowering pro-inflammatory markers like IL-1β and TNF-α. Overall, the findings suggest that Epicolin^®^ holds promise as an effective treatment strategy for glaucoma, addressing multiple underlying mechanisms contributing to RGC degeneration. This study aligns with discussions on the role of nutraceuticals in glaucoma management, as detailed in the previous paragraphs. Nutraceuticals, such as Epicolin^®^, leverage the therapeutic potential of natural compounds to provide neuroprotective effects, antioxidative activities, and anti-inflammatory benefits, which complement traditional IOP-lowering treatments. The synergistic action of the components within Epicolin^®^ exemplifies how multifactorial approaches can enhance the preservation of retinal health and function, addressing issues like oxidative stress and neuroinflammation that are prevalent in glaucoma. This reinforces the concept that integrating dietary supplements into glaucoma management strategies could offer tangible benefits for patient outcomes, particularly during the early stages of the disease.

Future studies should aim to optimize the dosage and treatment regimens for these nutraceuticals. Additionally, defining the exact molecular mechanisms by which these compounds exert their effects will be critical for understanding their potential as standalone therapies or in combination with traditional IOP-lowering medications. The integration of nutraceutical approaches in clinical practice may usher in a new paradigm in the treatment of glaucomatous conditions, offering a multifaceted strategy to combat both IOP elevation and the underlying neurodegenerative processes.

#### 3.3.8. Melatoninergic Compounds

The effects of agomelatine and melatonin on intraocular pressure (IOP) in glaucomatous subjects have been the focus of recent studies, highlighting their potential therapeutic roles in managing glaucoma. Agomelatine, a melatonin receptor agonist, was tested by us in a pilot study involving ten patients with hypertensive primary open-angle glaucoma (POAG) who were already receiving multiple topical hypotensive medications and in need of psychiatric treatment with agomelatine [120]. The results demonstrated a significant average reduction of approximately 30% in IOP after 15 and 30 days of daily oral administration of 25 mg of agomelatine, indicating its potential efficacy as a complementary treatment for patients who do not sufficiently respond to conventional therapies. This systemic administration suggests that agomelatine may provide an alternative pathway for IOP regulation through its melatoninergic action, thereby contributing to enhanced ocular health in glaucoma patients.

Therefore, we next investigated the hypotensive and neuroprotective effects of a topical nanomicellar formulation containing melatonin and agomelatine (MelAgo) in a rat model of hypertensive glaucoma induced by MCE [121,122]. The studies investigated a novel ophthalmic formulation containing melatonin (0.4%) and agomelatine (0.4%), stabilized with 11.5% Soluplus (a polymeric solubilizer) and 0.1% lipoic acid (to prevent oxidation). A pharmacokinetic analysis of healthy rats revealed that enough active molecules were retained in the eye after topical administration, with agomelatine showing better penetration into the posterior segment compared to melatonin. The concentrations achieved (equivalent to 17 nM melatonin and 378 nM agomelatine in the retina) were sufficient for receptor binding, with agomelatine’s higher retinal bioavailability suggesting enhanced tissue distribution. In the first study, the prolonged hypotensive effects of melatonin in nanomicelles compared to saline formulations was emphasized, revealing that both melatonin and agomelatine significantly lower IOP when applied separately or in combination. The addition of lipoic acid further extended the duration of these effects (up to 8 h) [121]. Conversely, the second study highlights not only the hypotensive effects but also the neuroprotective capabilities of the melatonin/agomelatine formulation. We compared MelAgo’s efficacy to standard glaucoma treatments (timolol and brimonidine), measuring IOP, RGC survival, inflammatory markers, and retinal function via ERG [122]. The nanomicellar system prolonged the corneal residence time, enabling sustained drug release, and the three-dose regimen (40 µg/eye every 2 h) ensured therapeutic levels. In the hypertensive glaucoma model, this formulation achieved a 60% IOP reduction—twice that of timolol or brimonidine—alongside significant neuroprotection, suppressing gliosis and apoptosis. The results highlight the formulation’s dual advantages, optimized ocular pharmacokinetics and potent therapeutic effects, positioning it as a promising glaucoma therapy candidate.

Following the exploration of agomelatine associated with melatonin, further research focusing on melatonin itself has revealed its multifaceted role in managing glaucomatous conditions, particularly through neuroprotective mechanisms [123]. Melatonin’s antioxidant attributes and ability to modulate neuroinflammatory responses contribute to its protective effects on RGCs by maintaining cellular homeostasis in the retina. Furthermore, its application as eye drops enhances local bioavailability, overcoming challenges associated with aqueous solubility and ensuring effective delivery to the ocular tissues. This dual action—lowering IOP and providing neuroprotection—positions melatonin as a promising candidate for glaucoma treatment. Finally, in addition to lowering IOP and protecting RGCs, melatonin eye drops offer several benefits for ocular surface health and the lens [123]. The application of melatonin can enhance tear production and stabilize the tear film, which may alleviate symptoms associated with dry eye conditions. Furthermore, melatonin’s antioxidant effects can help in mitigating oxidative damage to the lens, potentially delaying the onset of cataracts. The ability to improve ocular surface integrity not only contributes to patient comfort but also enhances the efficacy of other therapies being used to manage glaucoma and other ocular conditions. Overall, both agomelatine and melatonin present a multifaceted approach to treating glaucomatous damage, combining IOP reduction with neuroprotection and broader benefits for ocular health, underscoring the need for further investigation into their clinical applications.

## 4. Other Retinopathies

The study of retinopathies such as age-related macular degeneration (AMD), diabetic retinopathy, and retinopathy of prematurity (ROP) relies heavily on preclinical models that replicate key features of human disease. As highlighted in recent work [124], animal models remain indispensable for bridging mechanistic discoveries to clinical applications. For example, oxygen-induced retinopathy (OIR) models mimic ischemic proliferative retinopathies like ROP, while light-damage paradigms recapitulate oxidative stress in AMD. These systems have been instrumental in validating therapies such as Uparant (Section 4.1) and antioxidant formulations (Section 4.2.3). Similarly, in glaucoma research, chronic intraocular pressure (IOP) elevation models and optic nerve crush (ONC) have been critical for testing neuroprotective agents (Section 3.3). This translational foundation underscores the importance of model-informed therapeutic development, which now extends to emerging strategies for retinal vascular and degenerative diseases.

Retinopathies such as age-related macular degeneration (AMD), diabetic retinopathy, and retinopathy of prematurity (ROP) pose significant public health challenges, impacting millions of individuals worldwide and resulting in substantial visual impairment and blindness. AMD is the leading cause of vision loss among older adults, characterized by the degeneration of macular photoreceptors and retinal pigment epithelium, leading to central vision loss [125]. Diabetic retinopathy, a microvascular complication of chronic hyperglycemia often exacerbated by systemic hypertension [126], is characterized by progressive damage to retinal vasculature, pathological neovascularization, and eventual vision loss. It remains a leading cause of preventable blindness in the working-age population [127]. Conversely, ROP primarily affects premature infants and results from abnormal retinal blood vessel growth, which, if not addressed promptly, can lead to severe visual impairment [128].

The diagnosis and monitoring of retinopathies have traditionally relied on imaging techniques like optical coherence tomography (OCT) and fluoroangiography, as well as clinical examinations. Additionally, recent research highlights the potential of electrophysiological methods, particularly flicker electroretinography (ERG), to provide objective and quantifiable measures of retinal dysfunction. We have recently shown that the implicit time of the flicker ERG B-wave is a highly reliable parameter for detecting photoreceptor damage across various retinopathies, including AMD, diabetic retinopathy, and hypertensive retinopathy [129]. Our findings revealed that implicit time values exhibit minimal variability and no overlap between healthy and pathologic subjects, unlike amplitude measurements, which showed significant variability and overlapping ranges. This suggests that implicit time could serve as a robust diagnostic tool, offering a functional assessment of retinal health that complements structural imaging. Furthermore, our study underscored the independent progression of retinopathies in each eye, as evidenced by the lack of a correlation between flicker ERG parameters in affected eyes. By integrating such electrophysiological insights with existing diagnostic approaches, clinicians may enhance their ability to detect and monitor retinopathies early, ultimately improving patient outcomes and mitigating the global burden of these debilitating conditions. A very recent synthesis, featured in a special issue I co-edited [130], illustrates how converging advances in diagnostic technologies, genetic insights, and multimodal therapies are transforming retinopathy care. This paradigm shift encompasses everything from AI-powered early detection to innovative combination therapies that simultaneously target both vascular and neurodegenerative aspects of the disease.

Neovascularization, or pathological angiogenesis, is the abnormal and excessive growth of new blood vessels in the retina, particularly in the macular region. This process is a hallmark of the most severe and vision-threatening retinopathies, including ROP, proliferative diabetic retinopathy, and wet age-related macular degeneration [131]. This phenomenon leads to local leakage due to the increased permeability of the newly formed vessels, ultimately causing retinal toxicity, detachment, and, in many cases, blindness. As a result, antiangiogenic treatments, mostly derived from cancer therapies, have become the primary strategy to counteract this shared pathological event.

Despite these efforts, traditional treatments have had limited success in halting disease progression and improving patient outcomes, highlighting the need for innovative medications and nutraceuticals. Emerging treatments show promise in addressing the underlying mechanisms of these retinopathies. Additionally, specially formulated nutraceuticals that incorporate bioactive compounds with antioxidant and anti-inflammatory properties offer a potential approach to prevent or alleviate retinal degeneration. These advancements present an opportunity to enhance patient care and outcomes by offering multifaceted treatment strategies aimed at preserving vision and improving the quality of life for those affected by these prevalent retinopathies [132].

### 4.1. Uparant

The development of Uparant (acronym for Urokinase Plasminogen Activator Receptor Antagonist) marks a significant breakthrough in targeting pathological neoangiogenesis, particularly in conditions like cancer and chronic inflammation where excessive blood vessel formation occurs. Originally designed as a synthetic peptide antagonist of the urokinase plasminogen activator receptor (uPAR), Uparant was developed through a systematic investigation of uPAR’s role in cell migration and angiogenesis.

Key to this development was the identification of the critical chemotactic sequence Ser88-Arg-Ser-Arg-Tyr92 (SRSRY) within uPAR, which mediates endothelial cell signaling and migration essential for angiogenesis [133,134]. Subsequent research revealed this sequence promotes angiogenesis through protease-independent mechanisms, primarily via an interaction with the formyl peptide receptor (FPR).

This discovery led to the creation of competitive inhibitory peptides, including pERERY-NH2 (pyroglutamic acid-Arg-Glu-Arg-Tyr-NH2), designed to block SRSRY–FPR binding; and RERF (Arg-Glu-Arg-Phe-NH2), an optimized peptide derivative showing enhanced antiangiogenic properties.

RERF specifically demonstrated the potent inhibition of endothelial tube formation and VEGF-induced responses [135,136], establishing uPAR-derived peptides as promising antiangiogenic agents. These findings provided the foundation for Uparant’s development as a targeted therapeutic approach.

Building on these findings, researchers developed optimized analogues of the RERF peptide, culminating in the creation of Uparant (Ac-L-Arg-Aib-L-Arg-D-Cα(Me)Phe-NH2). This novel compound preserves the critical structural features required for biological activity while demonstrating enhanced metabolic stability and pharmacological potency. Uparant’s unique β-turn conformation confers resistance to enzymatic degradation and tolerance to sterilization processes, making it particularly suitable for clinical development.

Mechanistically, Uparant functions as a competitive inhibitor of the formyl peptide receptor (FPR), effectively displacing the natural ligand fMLF. It demonstrates potent antiangiogenic activity by blocking VEGF-induced endothelial cell migration and disrupting the cytoskeletal reorganization essential for neovascularization [137]. Preclinical studies have validated Uparant’s therapeutic potential. The compound significantly inhibits VEGF-mediated capillary sprouting in vivo and completely prevents pathological neovascularization in rabbit models [137]. These findings position Uparant as a promising candidate for treating angiogenesis-dependent disorders, including cancer and inflammatory diseases.

By specifically targeting uPAR–FPR signaling pathways, Uparant represents a paradigm shift in angiogenesis modulation, offering new therapeutic opportunities for conditions characterized by aberrant vascular proliferation.

#### Uparant for Retinal Neoangiogenesis Diseases

**Figure 3:**
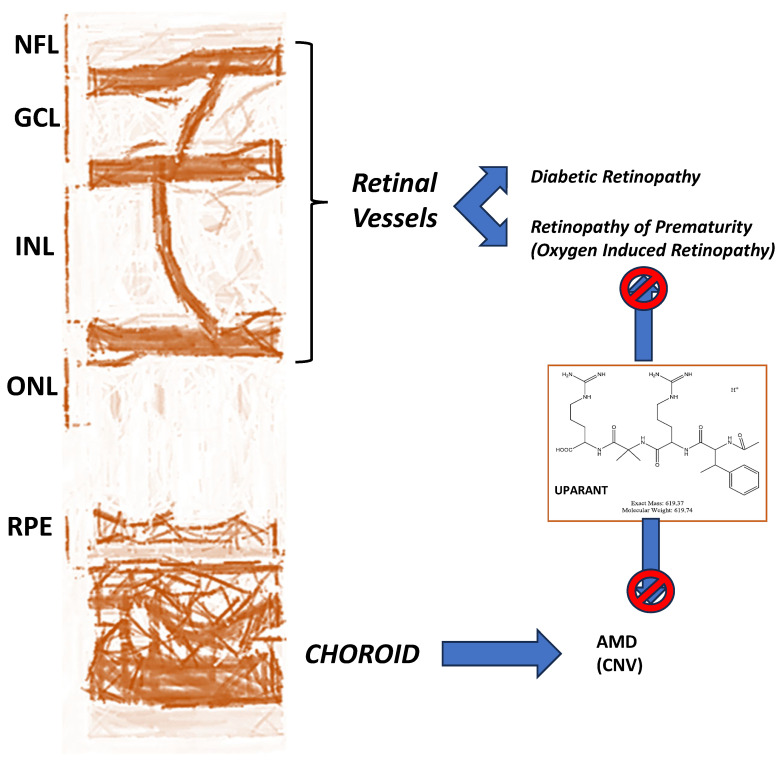
Uparant effect on neovascular retinopathies. The left panel illustrates the distribution of retinal blood vessels and the associated pathologies resulting from their degeneration. The right panel shows the effects of Uparant treatment in animal models of these conditions.

Building on the findings regarding Uparant’s antiangiogenic effects and molecular mechanisms in neovessel formation, we explored its potential as a therapeutic agent against retinal neovascularization (Figure 3). Our initial in vitro studies demonstrated that Uparant, at low nanomolar concentrations (1–100 nM), significantly inhibited VEGF-A-induced endothelial cell migration, invasion, and tube formation without compromising cell viability. This selectivity underscores Uparant’s promise as a targeted therapy. The study also revealed that Uparant disrupts downstream VEGF signaling, inhibiting the phosphorylation of VEGFR-2 and STAT3, leading to decreased expression of hypoxia-inducible factor 1-alpha (HIF-1α) and VEGF, indicating a feedback inhibition mechanism. Additionally, Uparant restored the blood–retinal barrier (BRB) integrity by preventing the VEGF-induced loss of tight junction proteins (claudin-1, claudin-5, and occludin) and normalizing transendothelial electrical resistance (TEER), highlighting its role in modulating angiogenesis and vascular permeability in retinal diseases [138].

Following these in vitro results, we assessed Uparant’s in vivo efficacy in a mouse model of oxygen-induced retinopathy (OIR), a surrogate for ischemic retinopathies like retinopathy of prematurity (ROP) [139]. The intravitreal administration of Uparant at postnatal days 12 and 15 significantly reduced neovascular tuft formation in the midperipheral retina while sparing the central avascular area, indicating the targeted inhibition of pathological angiogenesis. The study confirmed reduced levels of VEGF and phosphorylated VEGFR-2, along with the downregulation of HIF-1α and STAT3, consistent with earlier in vitro findings. Importantly, Uparant improved BRB integrity, as evidenced by increased occludin levels and reduced albumin leakage. It also enhanced retinal function, reflected by the restored electroretinography (ERG) amplitudes, and demonstrated anti-inflammatory effects by significantly lowering elevated inflammatory markers, such as TNF-α and IL-1β, in OIR retinas. Furthermore, Uparant countered the angiogenesis induced by vitreous fluid from PDR patients, indicating mechanisms beyond VEGF blockade, potentially involving uPAR-mediated integrin and formyl peptide receptor (FPR) signaling pathways [139].

We then investigated the efficacy of Uparant as an antiangiogenic and anti-inflammatory agent in a mouse model of laser-induced choroidal neovascularization (CNV), relevant to age-related macular degeneration (AMD) [140]. The findings aligned well with the previous results, revealing that intravitreal injections of Uparant significantly reduced the CNV area and leakage in the choroid, with effects comparable to those of established anti-VEGF therapies like bevacizumab. Importantly, Uparant was shown to mitigate the upregulation of the transcription factors HIF-1α and STAT3, which are crucial for the expression of angiogenic and inflammatory factors, thus normalizing their elevated levels after CNV induction. Moreover, we observed that while the uPA/uPAR/FPR system was upregulated in CNV, Uparant did not directly affect its expression, suggesting that its therapeutic benefits may arise from broader modulation of angiogenic and inflammatory pathways. Overall, these studies confirmed Uparant as a promising novel treatment for ocular neovascular conditions, potentially addressing the multifactorial nature of diseases like AMD more effectively than conventional therapies.

Next, we addressed the therapeutic potential of Uparant in treating diabetic retinopathy (DR) using two different diabetic rat models. A first study examined the effects of Uparant in spontaneously diabetic Torii (SDT) rats, demonstrating that the administration of Uparant prevented the deterioration of retinal function, reduced the levels of inflammatory and proangiogenic factors, and preserved blood–retinal barrier (BRB) integrity [141]. Specifically, Uparant mitigated electroretinogram (ERG) dysfunction and inhibited the upregulation of vascular endothelial growth factor (VEGF) and glial fibrillary acidic protein (GFAP), signaling a decrease in inflammation and gliosis associated with DR. A second study explored Uparant in streptozotocin-induced diabetic rats, where it was found to significantly restore ERG amplitudes, recover BRB integrity, and improve retinal function after treatment [142]. Uparant not only targets uPA/uPAR signaling pathways to combat pathology but also demonstrates a favorable pharmacokinetic profile, maintaining effective concentrations in the eye and retina for prolonged periods, suggesting its potential as a novel systemic therapeutic for early DR progression. Overall, both studies underscored Uparant’s dual role in anti-inflammatory and antiangiogenic mechanisms, presenting it as a promising therapeutic candidate for managing early stages of diabetic retinopathy.

In a final study, the critical role of inflammation in driving angiogenesis in proliferative diabetic retinopathy (PDR) and the unique ability of Uparant to counteract it was addressed [143]. The study demonstrated that the vitreous humor from PDR patients activates endothelial cells, promoting proliferation, migration, and neovessel formation, while also inducing a pro-inflammatory phenotype marked by leukocyte recruitment and inflammatory mediator release. These effects are mediated through N-formyl peptide receptors (FPRs), as evidenced by the correlation between CD45+ leukocyte infiltration and neovascularization. A key finding of the study is the differential efficacy of therapeutic agents: while classical anti-VEGF treatments (e.g., bevacizumab) showed limited effectiveness in inhibiting the endothelial proliferation induced by PDR vitreous, Uparant significantly suppressed both angiogenic and inflammatory responses. This confirms that Uparant has a broader mechanism of action compared to anti-VEGF therapies, targeting the inflammatory pathways that contribute to neovascularization in PDR. The study highlights the potential of Uparant as a dual anti-inflammatory and anti-angiogenic therapy for PDR, addressing the limitations of current VEGF-focused treatments. By targeting the interplay between inflammation and angiogenesis, Uparant may offer a more comprehensive approach to managing complex ocular conditions, also including retinitis pigmentosa [144,145].

### 4.2. Food Supplements for Ophthalmic Diseases

Uparant represents a new class of drugs designed for systemic administration via injection—either intravitreal or even subcutaneous. It was developed as a therapeutic treatment to be administered at the onset or during the progression of an overt retinal disease. In contrast, food supplements were studied and developed as ancillary treatments, intended for use when there was a high risk of developing a retinal pathology or at its earliest symptoms [132] (Table 1).

#### 4.2.1. Fatty Acid Group (FAG)

Inflammatory processes play a central role in the pathogenesis of retinal diseases, particularly AMD [154]. Macrophages, as key regulators of inflammation, demonstrate remarkable plasticity by transitioning between pro-inflammatory (M1) and anti-inflammatory (M2) phenotypes in response to microenvironmental signals. In chronic inflammatory conditions like AMD, this regulatory balance becomes disrupted, with dysregulated macrophage activity contributing to tissue damage through multiple mechanisms: excessive cytokine production, oxidative stress generation, and complement system activation [155].

Recent research highlights the potential of fatty acids to influence macrophage polarization dynamics. Specifically, specialized formulations such as the Fatty Acid Group (FAG^®^) composition have shown promise in modulating this phenotypic switching, suggesting a novel therapeutic approach to reestablish immune homeostasis in retinal disorders [156]. This immunomodulatory strategy may offer targeted intervention against the chronic inflammatory component of AMD and related retinal pathologies.

The dynamic interplay between macrophage phenotypes plays a pivotal role in inflammatory processes within retinal diseases. When polarized to the M1 state by stimuli such as lipopolysaccharides (LPS) or interferon-gamma (IFN-γ), macrophages drive inflammatory cascades through the robust secretion of cytokines including TNF-α, IL-6, and IL-8. In striking contrast, their M2 counterparts orchestrate tissue repair and inflammation resolution by producing anti-inflammatory mediators like IL-10 and TGF-β [157]. This delicate equilibrium assumes particular significance in age-related macular degeneration (AMD), where chronic low-grade inflammation creates a permissive environment for progressive retinal degeneration [125,154,155].

The modulation of macrophage behavior by fatty acids occurs through a sophisticated network of mechanisms that collectively influence inflammatory responses. By altering the membrane composition and fluidity, fatty acids can reshape cellular responsiveness to microenvironmental signals. Simultaneously, they exert profound effects on receptor-mediated signaling pathways and cellular metabolism. Among these lipid mediators, omega-3 fatty acids such as eicosapentaenoic acid (EPA) and docosahexaenoic acid (DHA) stand out for their ability to promote the beneficial M2 phenotype through dual modulation of inflammatory pathways—suppressing NF-κB activation while enhancing PPAR-γ signaling, resulting in attenuated pro-inflammatory cytokine production. Other fatty acids including palmitic and oleic acids contribute to inflammatory regulation by fine-tuning NLRP3 inflammasome activity and oxidative stress responses, while azelaic acid extends the anti-inflammatory arsenal by specifically inhibiting neutrophil chemotaxis [158]. This multifaceted regulation of macrophage polarization by fatty acids underscores their therapeutic potential in managing the chronic inflammatory components of retinal degeneration.

The therapeutic potential of FAG^®^ in AMD finds strong support in our preclinical investigations using a well-established mouse model of dry AMD induced by the subretinal injection of polyethylene glycol (PEG)-400. This model reliably reproduces key features of human AMD, including the formation of drusen-like deposits, oxidative stress, chronic inflammation, and progressive thinning of the outer retinal layers—all hallmarks of the disease that contribute to vision loss [146]. Our findings demonstrate that FAG^®^ supplementation produces multifaceted protective effects: it significantly reduces levels of pro-inflammatory cytokines (IL-6, TNF-α) while preventing the characteristic photoreceptor degeneration observed in this model, as quantified through preservation of the outer nuclear layer (ONL) thickness. Notably, FAG^®^ administration markedly decreases pathological macrophage infiltration (CD68+, F4/80+ cells) in the choroid—a pathological process known to contribute to both dry and wet AMD progression. While the current study did not specifically evaluate complement inhibition or oxidative stress parameters, the antioxidant components of FAG^®^ (including lycopene) suggest an additional plausible mechanism through which this formulation may protect against oxidative retinal damage. These collective findings position FAG^®^ as a promising multimodal intervention for AMD, potentially addressing both inflammatory and degenerative aspects of the disease.

These findings are corroborated by further research in which we used neuroinflammatory models. In this research, we found that FAG supplementation in experimental autoimmune encephalomyelitis (EAE)—a model of optic neuritis and multiple sclerosis—promotes a shift from pro-inflammatory M1 to anti-inflammatory M2 macrophage polarization, rescues retinal ganglion cell (RGC) degeneration, and restores optic nerve histopathology [147]. Notably, FAG’s efficacy in EAE extends to functional visual recovery, as evidenced by improved photopic electroretinogram (ERG) responses, while simultaneously preserving the neuroprotective role of macrophage-derived factors like oncomodulin (OCM) [148]. Together, all these studies highlight FAG’s dual capacity to dampen inflammation (via NF-κB suppression, PPAR-γ activation, and M2 polarization) and protect neuronal structures, offering a compelling rationale for its therapeutic potential in AMD and other inflammatory retinal diseases.

The clinical validation of specialized formulations like Macular-FAG^®^—which combines fatty acids with lycopene and spirulina—remains essential to substantiate these promising preclinical findings. While the AREDS2 study challenged the efficacy of isolated omega-3 supplementation [159], the sophisticated composition of FAG^®^ may offer distinct advantages. Its balanced omega-3/omega-6 ratio creates a more physiologically relevant lipid milieu that could enhance both anti-inflammatory and neuroprotective effects compared to single compounds. Future investigations should focus on three key areas: (1) optimizing FAG formulations for maximal bioavailability and tissue targeting, (2) exploring potential synergies with established therapies such as anti-VEGF agents or complement inhibitors, and (3) developing personalized treatment algorithms based on individual genetic risk profiles.

In summary, FAG^®^ embodies a comprehensive therapeutic strategy for retinal pathologies by simultaneously addressing multiple disease mechanisms—chronic inflammation, oxidative damage, and immune dysfunction. Its unique capacity to modulate macrophage polarization while potentially influencing complement activation makes it particularly relevant for the entire AMD spectrum, from dry to wet forms. These mechanistic advantages, coupled with its favorable safety profile, strongly justify accelerated clinical translation to determine its efficacy in preserving visual function and halting disease progression across different AMD stages.

#### 4.2.2. Calanus Oil

The therapeutic potential of fatty-acid-based interventions lies in their unique capacity to target the interconnected metabolic and inflammatory pathways underlying retinal diseases. Our research [160] revealed how next-generation formulations can transcend the limitations of conventional supplements through innovative sourcing and composition. Our work has particularly focused on Calanus oil, a novel marine-derived source of omega-3 fatty acids packaged in wax esters, which demonstrates superior bioavailability compared to conventional fish oil. When combined with potent xanthophyll antioxidants like astaxanthin, this formulation creates a multifaceted therapeutic platform that simultaneously addresses oxidative stress, inflammation, and metabolic dysregulation in retinal diseases.

Using the well-validated PEG-400 retinal degeneration model, we conducted a rigorous comparative study of these distinct lipid sources. The results were striking: the Calanus oil formulation consistently outperformed fish oil across all evaluated parameters. Molecular analyses revealed its superior capacity to modulate oxidative stress pathways, with more pronounced reductions in NQO-1 and HO-1 expression. Similarly, it demonstrated enhanced anti-inflammatory effects, showing greater suppression of NF-κB activation and IL-6 production. These biochemical advantages translated to measurable histological protection, including better preservation of outer nuclear layer thickness and attenuated glial activation—evidence of its multifaceted retinal protection.

The enhanced efficacy of the Calanus oil formulation likely stems from several unique properties. Unlike conventional fish oil, Calanus oil contains omega-3 fatty acids in wax ester form, which may improve bioavailability and tissue uptake. Additionally, the presence of policosanols and stearidonic acid (SDA), a metabolic precursor to EPA and DHA, may provide more efficient conversion to bioactive fatty acids in retinal tissues. The inclusion of astaxanthin, a potent antioxidant not present in the fish oil formulation, probably contributed to the superior oxidative stress protection observed with Calanus oil treatment. This study highlights how fundamental differences in the lipid composition can profoundly impact therapeutic efficacy. The unique structural and biochemical properties of Calanus oil—particularly its wax ester-bound omega-3 fatty acids, long-chain policosanols, and endogenous astaxanthin—create an integrated biological matrix that appears uniquely capable of addressing the multifactorial pathogenesis of retinal degeneration. These compositional advantages translate to superior bioavailability and enhanced cellular protection, positioning Calanus oil as a potentially more effective therapeutic option than conventional fish oil for preserving retinal structure and function.

These preclinical findings acquire greater clinical relevance when contextualized with observations from AMD patient studies. While the AREDS2 trial demonstrated the limited efficacy of isolated omega-3 supplementation, our results indicate that advanced formulations combining bioavailable fatty acids with targeted antioxidants may offer superior therapeutic benefits. The PEG-400 model’s unique capacity to recapitulate both the inflammatory and degenerative features of AMD enhances the translational potential of these findings. Importantly, our work emphasizes that therapeutic efficacy depends not merely on the presence of active compounds, but on their formulation, bioavailability, and synergistic interactions within complex biological systems.

The implications of this research extend to several promising directions for future investigation. Subsequent studies should focus on establishing optimal dosing protocols, evaluating long-term effects in chronic degeneration models, and exploring potential synergistic effects with current AMD therapies. The marked differences between Calanus oil and conventional fish oil formulations underscore the potential for personalized treatment strategies based on genetic predisposition and disease progression. As research continues to elucidate the intricate relationships between retinal lipid metabolism and inflammatory pathways, targeted fatty acid-based therapies may become increasingly valuable in clinical practice for preventing vision loss in AMD and related conditions. Our study [160] establishes a robust framework for this translational development, demonstrating how rationally designed nutraceutical formulations can simultaneously target multiple pathological mechanisms in retinal degeneration.

#### 4.2.3. The Role of Antioxidants

Cyanidin-3-O-glucoside (C3G), lutein, zeaxanthin, and astaxanthin are potent bioactive compounds with significant antioxidant properties that play crucial roles in protecting retinal cells from oxidative stress, a key factor in the pathogenesis of retinal diseases. These compounds mitigate oxidative damage through multiple mechanisms, including the direct scavenging of reactive oxygen species (ROS), modulation of endogenous antioxidant pathways, and preservation of cellular integrity.

C3G, an anthocyanin found in dark-colored fruits and vegetables, has demonstrated remarkable efficacy in counteracting high glucose-induced oxidative stress in retinal endothelial cells. In a pioneering in vitro study [149], we have highlighted its ability to restore cell viability, reduce ROS levels, and maintain the integrity of the blood–retinal barrier (BRB) by upregulating tight junction proteins like ZO-1 and VE-cadherin. Its protective effects are further enhanced when combined with verbascoside (a bioactive phenylpropanoid glycoside found in many medicinal plants, known for its potent antioxidant, anti-inflammatory, and neuroprotective properties), showcasing synergistic antioxidant and anti-inflammatory actions. C3G also activates pathways like GSH synthesis and eNOS, improving vascular function and reducing oxidative damage in retinal tissues [161].

Lutein and its stereoisomer zeaxanthin are xanthophyll carotenoids concentrated in the macula, where they form the macular pigment. These compounds absorb high-energy blue-violet light, shielding photoreceptors and retinal pigment epithelium (RPE) cells from phototoxic damage. Their antioxidant properties involve quenching singlet oxygen and neutralizing free radicals, thereby preventing lipid peroxidation and mitochondrial dysfunction [162]. We conducted in vitro research using our own cell line [150], demonstrating that lutein effectively reduces ROS production and apoptosis in corneal cells—and likely retinal cells—exposed to blue light. Additionally, we found that lutein and zeaxanthin modulate the Nrf2 pathway, increasing the expression of antioxidant enzymes such as heme oxygenase-1 (HO-1), thereby enhancing cellular defense mechanisms.

Astaxanthin, another potent carotenoid, exhibits superior antioxidant activity due to its unique molecular structure, which enables it to span cell membranes and neutralize ROS at multiple sites. Studies demonstrate its protective effects on retinal cells by inhibiting oxidative-stress-induced apoptosis and inflammation, particularly in models of UV and blue light exposure [163]. Furthermore, astaxanthin activates the Nrf2/HO-1 pathway and suppresses NF-κB signaling, highlighting its dual role in mitigating oxidative and inflammatory damage [150]. Although its absorption spectrum is slightly redshifted compared to lutein, astaxanthin complements lutein’s effects, offering broader protection across the light spectrum.

Together, these compounds offer a multi-faceted defense against oxidative stress in retinal cells. Their combined use could enhance protection by targeting different oxidative pathways and light wavelengths. For instance, lutein and zeaxanthin are more effective against short-wavelength blue light, while astaxanthin provides additional coverage for longer wavelengths. This integrative approach is particularly relevant in clinical settings, where dietary supplementation or even topical formulations of these antioxidants could delay or prevent the progression of retinal diseases, offering a non-invasive therapeutic strategy alongside conventional treatments. Further research is needed to optimize their bioavailability and synergistic effects, but current evidence strongly supports their role in preserving retinal health through oxidative stress mitigation.

The protective effects of C3G in association with lutein, verbascoside, and zinc have been demonstrated in preclinical studies using in vivo models of retinal damage [151]. We have used a rat model of light-induced retinal damage, in which we have shown that the combined administration of C3G and lutein significantly reduced oxidative stress markers such as Nrf2 and HO-1, attenuated inflammatory responses by lowering NF-κB and IL-6 levels, and prevented gliosis and microglial activation, as evidenced by decreased GFAP and Iba-1 immunoreactivity. This combination also preserved photoreceptor integrity, as shown by maintained outer nuclear layer thickness and rhodopsin expression, and improved retinal function, with electroretinography (ERG) recordings revealing restored scotopic and photopic responses. The efficacy of C3G was further enhanced when incorporated into a multicomponent mixture that included verbascoside and zinc, which collectively provided synergistic antioxidant and anti-inflammatory effects, offering superior protection compared to individual compounds alone [151].

Similarly, in a streptozotocin-induced diabetic retinopathy model, we demonstrated that a formulation containing C3G, verbascoside, and zinc exerted dose-dependent protective effects [152]. The treatment reduced oxidative stress by lowering ROS levels and normalizing Nrf2/HO-1 expression while suppressing inflammation through the inhibition of NF-κB and IL-6 pathways. These improvements were accompanied by enhanced blood–retinal barrier (BRB) function, evidenced by restored tight junction proteins (ZO-1 and Claudin-5) and reduced vascular leakage. The formulation also attenuated apoptosis, as shown by a decreased Bax/Bcl-2 ratio and reduced caspase-3 activation, while preserving retinal function. Electroretinography (ERG) revealed significant recovery of a- and b-wave amplitudes. Notably, the high-dose formulation was particularly effective, nearly normalizing retinal parameters to control levels. These findings underscore the potential of multicomponent antioxidant blends to address the multifactorial pathology of diabetic retinopathy [152].

These findings underscore the therapeutic potential of C3G in combination with other bioactive compounds, leveraging their complementary mechanisms to address oxidative stress, inflammation, and neurodegeneration in retinal diseases. The synergistic interactions among these compounds not only enhance their individual bioactivities but also improve their overall efficacy, providing a rationale for the development of nutraceutical formulations aimed at preventing or delaying the progression of retinal damage in conditions such as phototoxicity and diabetic retinopathy.

An important clinical confirmation of these findings emerged from our collaborative study with Dr. Filippello and colleagues [153], which demonstrated the protective effects of a specific antioxidant blend on retinal health under conditions of high oxidative stress from intense light exposure. Our formulation combining C3G, lutein, verbascoside, and zinc showed both cellular protection in vitro and measurable improvements in visual performance among individuals exposed to demanding light conditions, particularly sport motorcycle test pilots. In preliminary in vitro experiments, human retinal pigment epithelial (RPE) cells exposed to intense white light showed a dramatic increase in reactive oxygen species (ROS) and significantly reduced viability. Pretreatment with lutein and C3G—individually and in combination—effectively mitigated these effects. The combined treatment produced the most striking results, completely neutralizing oxidative stress and restoring cell viability, suggesting a synergistic interaction between these compounds [153]. Clinical observations strongly supported these laboratory findings. Test pilots who received the supplement for 70 days demonstrated significant improvements in multiple visual parameters. Objective measures showed enhanced far-sight visual acuity, better glare adaptation, faster recovery from bright light exposure, and improved twilight vision. The supplement also improved contrast sensitivity and stereoscopic perception—critical factors for depth judgment and obstacle detection. Subjectively, participants reported reduced eye fatigue and greater visual comfort in both daylight and low-light conditions, confirming the formulation’s practical benefits [153].

The success of this formulation can be attributed to the complementary mechanisms of its components. Lutein acts as a blue light filter and antioxidant [150,162], while C3G supports rhodopsin regeneration and activates cellular defense pathways like Nrf2 [149,161]. Verbascoside contributes anti-inflammatory and neuroprotective effects, and zinc bolsters enzymatic antioxidant systems [151,152]. Together, they create a robust shield against oxidative damage while promoting retinal function.

While the study’s retrospective nature and limited sample size call for further validation through larger, controlled trials, the results are highly encouraging. They suggest that targeted antioxidant supplementation can play a meaningful role in preserving retinal health, particularly for individuals exposed to extreme visual stressors—whether athletes, drivers, or professionals working in variable lighting conditions [153]. This research not only advances our understanding of retinal protection but also highlights a practical, nutraceutical approach to enhancing visual performance in high-demand settings. Ultimately, the findings support the idea that a well-designed antioxidant blend can bridge the gap between laboratory research and real-world benefits, offering a promising strategy to safeguard vision against the relentless challenges posed by light-induced oxidative stress [151,152].

#### 4.2.4. Myoops^®^

The studies by Amato et al. [151] and Filippello et al. [153] demonstrated the protective and performance-enhancing effects of antioxidant supplements on retinal function. Along the same line, however years before, we published a clinical observation with a different food supplement—Myoops^®^—containing a mix of vitamins and antioxidants. This study [164] investigated the impact of Night Vision Goggles (NVGs) on retinocortical bioelectrical activity and evaluated the potential of a food supplement to mitigate these effects. The research involved healthy male aircrew members who underwent Visual Evoked Potential (VEP) recordings under two conditions: unaided photopic (normal light) and mesopic (low light) with NVGs. The results demonstrated that NVG use significantly impaired VEP responses, increasing the latency and decreasing the amplitude, which are critical indicators of visual signal transmission efficiency. These findings suggest that NVGs, while enhancing visibility in low-light conditions, may introduce delays and reduce the strength of visual signals processed by the cortex. To counteract these effects, the study tested a food supplement containing anthocyanosides, procyanidolic oligomers, lutein, and vitamins A and E (Myoops^®^). After 45 days of supplementation, participants showed significant improvements in VEP parameters, with a reduced latency and increased amplitude, indicating enhanced foveal selectivity and photoreceptor sensitivity. Pescosolido’s work thus provides early evidence that antioxidant supplements can enhance visual functions under challenging conditions, such as low-light environments with NVGs. The study underscores the potential of these compounds to support retinal health and optimize visual processing, complementing later findings that emphasize their role in protecting against oxidative stress and improving visual acuity in both clinical and high-performance settings. Together, these studies reinforce the therapeutic promise of antioxidant-rich supplements in preserving and enhancing visual function across diverse conditions.

#### 4.2.5. Autophagy

In exploring innovative approaches to the prevention and treatment of diabetic retinopathy (DR), the role of cellular mechanisms such as autophagy has gained significant attention. We addressed the role of autophagy within the context of DR, highlighting its dualistic nature in cellular health and disease [165]. Autophagy is crucial for maintaining cellular homeostasis, particularly under the stress of chronic hyperglycemia, which is a hallmark of diabetes. This process allows cells to degrade and recycle damaged organelles and proteins, thereby fostering cell survival. However, when autophagy is dysregulated—often exacerbated by factors such as oxidative stress and inflammatory responses—it can lead to cellular dysfunction and increased apoptosis.

Diabetic retinopathy (DR) progression is characterized by microvascular alterations leading to retinal ischemia and increased vascular permeability, ultimately resulting in vision impairment [126,127]. Our findings highlight the critical role of the autophagy–apoptosis equilibrium in determining the retinal cell fate under diabetic conditions. This mechanistic interplay suggests that therapeutic strategies modulating autophagic pathways may offer promising interventions for DR management [165].

One of the innovative strategies proposed in the study involves the use of mTOR inhibitors, such as rapamycin, which could effectively modulate autophagy [165]. By enhancing the protective aspects of this process while inhibiting its damaging consequences, such treatments may control pathological neovascularization—a key factor in the progression of DR. In addition, we discuss the potential of combining autophagy modulation with anti-inflammatory treatments, thereby creating a comprehensive strategy to address not only the symptoms of DR but also the underlying mechanisms contributing to its development [143]. The integration of autophagy modulation into therapeutic strategies represents a promising advancement in diabetic retinopathy (DR) treatment. Our research highlights this paradigm shift toward targeting fundamental cellular processes to preserve vision in diabetic patients. These innovative approaches—whether through nutritional interventions or innovative pharmaceutical agents like Uparant—offer new possibilities for DR management by addressing the underlying retinal pathophysiology [132,141,142]. By precisely targeting these molecular mechanisms, such strategies may not only improve visual outcomes but also prevent long-term vision loss in diabetes.

#### 4.2.6. Hereditary Retinopathies

Hereditary retinopathies represent a group of eye disorders that are primarily caused by genetic mutations affecting the retina, leading to progressive visual impairment. One notable example is Leber’s hereditary optic neuropathy (LHON), a genetic condition resulting in severe vision loss due to the degeneration of RGCs. The treatment of LHON has traditionally relied on chronic high doses of oral antioxidants, such as idebenone, which aim to counteract the oxidative stress that contributes to ganglion cell death [166]. However, this approach often comes with significant side effects, including gastrointestinal issues and other systemic reactions, which can limit patient adherence and overall treatment effectiveness. Consequently, there is an urgent need for alternative treatment strategies that can enhance antioxidant delivery while minimizing these adverse effects. Recent research suggests that a novel topical application of antioxidants might provide a promising solution. By applying antioxidants directly to the ocular surface, this approach could potentially increase local concentrations at the retina, thereby maximizing therapeutic efficacy while allowing for lower overall doses. Such advancements could transform the management of LHON and other hereditary retinopathies, ultimately leading to improved visual outcomes and patient quality of life.

The search for more effective and tolerable antioxidant therapies in hereditary retinopathies such as LHON has led to innovative approaches that prioritize targeted delivery and enhanced bioavailability. Our studies on lipophilic derivatives of edaravone (C18Edv) offer compelling insights into how structural modifications and delivery systems can optimize antioxidant efficacy while mitigating systemic side effects. In the first work [167], we demonstrated that alkylating the potent antioxidant edaravone with a C18 hydrocarbon chain (C18Edv) significantly improved its affinity for lipid membranes, a critical factor for ocular drug delivery, where the retina’s high lipid content makes it particularly susceptible to oxidative damage. This lipophilic derivative not only retained the radical-scavenging activity of the parent compound but also exhibited superior protection against lipid peroxidation in retinal pigment epithelium (ARPE-19) cells, especially when oxidative stress was induced within the lipid bilayer. These findings underscore the potential of membrane-targeted antioxidants to address the oxidative stress driving retinal ganglion cell degeneration in LHON. Building on this, further studies [168,169] explored the formulation of C18Edv into liposomes, revealing that the antioxidant’s incorporation into 1-palmitoyl-2-oleoyl-sn-glycero-3-phosphocholine (POPC) bilayers—a common phospholipid used in model membranes—modulated membrane rigidity and hydration in a concentration-dependent manner. Notably, liposomes with 20% *w*/*w* C18Edv achieved an optimal balance between cellular uptake and antioxidant activity, outperforming both lower and higher concentrations. This precision in the formulation aligns with the need for topical therapies that can deliver high local concentrations of antioxidants to the retina without systemic exposure, thereby avoiding the gastrointestinal and other side effects associated with oral high-dose idebenone. Moreover, the liposomal encapsulation of C18Edv addresses the challenge of poor water solubility, a common limitation for lipophilic drugs, while enhancing stability and bioavailability at the ocular surface. Together, these studies highlight a promising pathway for developing topical antioxidant treatments: by leveraging lipophilic modifications and nanocarrier systems, it becomes possible to achieve therapeutic retinal concentrations with minimal off-target effects. Such advancements could revolutionize the management of LHON and related retinopathies, offering a paradigm shift from systemic to localized, patient-friendly therapies that preserve vision and improve quality of life.

## 5. Refractive Errors

Refractive errors represent one of the most widespread yet frequently overlooked public health challenges of our time. These vision disorders—myopia, hyperopia, astigmatism, and presbyopia—arise from imperfections in the eye’s ability to focus light precisely on the retina, leading to blurred vision at various distances. While easily correctable with glasses, contact lenses, or refractive surgery, their impact extends far beyond individual eyesight, touching nearly every aspect of human development, economic productivity, and social well-being.

The scale of the problem is staggering. According to the World Health Organization’s reports [170], an estimated 2.6 billion people worldwide live with uncorrected refractive errors, making this the leading cause of preventable vision impairment globally. Among these, some 514 million individuals suffer from moderate to severe distance vision impairment due to untreated myopia, hyperopia, or astigmatism, as documented in the Global Burden of Disease Study [171]. Additionally, a systematic review by Fricke et al. [172] reported that presbyopia significantly contributes to vision impairment, particularly among older populations. Mostly alarming is the rapid rise of myopia, which current epidemiological models predict will affect half the world’s population by 2050, as recently highlighted [173]. This myopia epidemic, driven largely by lifestyle changes including reduced outdoor activity and increased near work, carries significant long-term risks, including higher chances of developing sight-threatening complications like retinal detachment or glaucoma later in life.

The socioeconomic ramifications of uncorrected vision are profound. A 2019 analysis estimated that uncorrected myopia alone costs the global economy approximately $244 billion annually in lost productivity—a figure reflecting workforce participation declines rather than direct healthcare costs [174]. Refractive errors remain a significant barrier to education, particularly in underserved communities. In U.S. urban high-poverty settings, 35.5% of students failing school-based vision screenings had clinically significant refractive errors, with myopia and astigmatism disproportionately affecting Black and Latin students [175]. When left untreated, these conditions impair academic performance: a randomized trial in rural China demonstrated that providing free eyeglasses improved test scores by 0.16–0.22 standard deviations—equivalent to 0.3–0.5 additional years of schooling [176]. Despite this evidence, access to vision care remains limited in low-resource settings due to systemic gaps in cost, awareness, and cultural barriers. School-based programs effectively identify unmet need [175], but broader interventions are required to address this avoidable disability globally.

Beyond the economic impacts, the human costs of uncorrected vision impairment are profound, affecting nearly every dimension of the quality of life. The World Health Organization identifies uncorrected refractive errors as a modifiable risk factor for both road traffic accidents and reduced workplace productivity, particularly in low-resource settings [170]. These findings support the WHO’s position that accessible vision care is essential for workforce development and public safety.

The psychosocial consequences are equally significant. Multiple studies demonstrate that vision impairment increases risks of depression, social isolation, and loss of independence, especially among older populations. A U.S. study during COVID-19 found that adults with visual impairment had 1.5–2.0 times higher odds of depression and anxiety compared to those with normal vision [177]. These effects compound the daily challenges of impaired mobility and reduced occupational capacity.

Refractive errors remain among the most tractable public health challenges, with the World Health Organization estimating that 80% of cases could be resolved through affordable interventions—primarily corrective lenses. Evidence-based solutions exist: systematic reviews demonstrate that national school vision screening programs in low- and middle-income countries (LMICs) effectively reduce uncorrected refractive errors in children, significantly improving visual outcomes [178]. These programs emphasize early detection, which may help slow myopia progression and address this preventable cause of vision impairment.

Emerging research highlights modifiable risk factors, with meta-analyses of randomized controlled trials confirming that increased outdoor time significantly reduces myopia incidence in children [179]. As digitalization intensifies visual demands in education and workplaces, ensuring universal access to vision correction becomes both a medical priority and socioeconomic imperative. The documented benefits span educational attainment, workforce productivity, and quality of life—making equitable implementation of these proven, cost-effective solutions an urgent global health priority.

### 5.1. Myopia

Myopia, or nearsightedness, is a prevalent refractive error characterized by the inability to see distant objects clearly, due to an elongation of the eyeball or excessive curvature of the cornea. The development of myopia is a complex interplay of genetic and environmental factors, with increasing evidence suggesting that prolonged near work and reduced exposure to outdoor light significantly contribute to its progression [180]. Studies indicate that myopia often begins in childhood and can worsen during periods of rapid eye growth, leading to high myopia [181], which is defined as a refractive error greater than −6.00 diopters (D). This condition not only compromises visual acuity but also increases the risk of serious ocular complications, including retinal detachment, glaucoma, and myopic macular degeneration in later life [182].

The use of low-dose atropine eye drops has emerged as a promising therapeutic strategy for both the treatment and prevention of high myopia in children [183]. Atropine, an anticholinergic agent, works primarily by blocking the action of acetylcholine, reducing the eye’s accommodative response, which is thought to play a role in myopia progression. The physiological mechanism behind atropine’s efficacy involves its ability to temper excessive eye growth by modulating the signaling pathways that regulate axial length, thereby slowing the elongation of the eyeball [184].

Preclinical studies have demonstrated that low-dose atropine effectively reduces the progression of myopia in animal models. For instance, experiments on chick and monkey models have shown that atropine administration leads to a significant decrease in axial elongation and a corresponding reduction in myopic development [185]. These findings have paved the way for clinical trials on human subjects, which have consistently shown that low-dose atropine (commonly at concentrations of 0.01% to 0.1%) slows myopia progression more effectively than a placebo, with minimal side effects [186].

In conclusion, the integration of low-dose atropine into the management of high myopia in children not only addresses the immediate concerns of vision correction but also represents a proactive measure to mitigate the long-term risks associated with severe myopia. Additionally, research suggests that 7-methylxanthine (7MX)—an active metabolite of caffeine and theobromine—may also exhibit similar effects in controlling myopia development and progression, providing further options for managing this growing public health concern [187]. As the prevalence of myopia continues to rise globally, the use of these pharmacological interventions, alongside behavioral modifications such as increased outdoor activity, could significantly reshape the landscape of myopia prevention and treatment in pediatric populations.

#### 5.1.1. In Vitro Atropine and 7MX Effects

Intrigued by these results, and considering that eye growth is primarily regulated at the level of the sclera [188], we decided to investigate the role of atropine and 7MX in controlling eye growth and myopia using an in vitro model system with scleral and choroidal fibroblasts. Building upon our understanding of myopia management through pharmacological interventions, our findings [189] offer significant insights into the dual role of atropine and 7MX in modulating eye growth. Our study addressed how atropine influences the production of extracellular matrix (ECM) components in both scleral and choroidal fibroblasts, thereby impacting the scleral rigidity and overall eye morphology. Importantly, we demonstrated that atropine enhances the biosynthesis of collagen type I and fibronectin in human scleral fibroblasts, supporting the hypothesis that strengthening the scleral ECM can retard the axial elongation associated with myopia progression. This mechanism aligns with the prevailing theories regarding myopia pathology, where scleral thinning correlates with excessive eye elongation [190]. Equally, our study reveals that 7MX also promotes collagen production in scleral fibroblasts, providing further evidence of its potential as a therapeutic agent against myopia. However, both atropine and 7MX exhibited a contrasting inhibitory effect on choroidal fibroblasts, reducing their ECM production. This reduction in the ECM density could potentially lead to improved blood perfusion within the choroid by increasing vascular permeability. Enhanced perfusion is crucial for maintaining optimal metabolic conditions in the eye, as adequate blood supply ensures efficient nutrient and oxygen delivery while facilitating the removal of metabolic waste. Importantly, choroidal thickening and blood flow are closely correlated with the regulation of scleral growth. By thinning the ECM in choroidal fibroblasts, atropine and 7MX might mitigate scleral hypoxia—a condition associated with excessive myopic eye elongation [191,192].

Thus, this mechanism not only reinforces the multifaceted roles of these pharmacological agents but also suggests that balancing ECM dynamics in both the sclera and choroid could be a viable strategy for controlling myopia progression. Moreover, the investigation highlights critical considerations regarding the ocular surface toxicity of atropine, noting that prolonged treatment can lead to corneal epithelial cell damage, an issue that may limit its long-term use in pediatric patients [193]. Fortunately, the study also presents promising solutions, indicating that the combined use of atropine with bioactive compounds like colostrum or 2-fucosyl-lactose can mitigate these toxic effects and enhance cell viability [189]. This finding not only solidifies the importance of atropine and 7MX as viable options in controlling myopia but also opens avenues for improving treatment regimens, emphasizing the necessity of balancing efficacy with safety in managing this increasingly prevalent vision disorder among children.

#### 5.1.2. Proposals for Coadjuvant Treatments of Progressive Myopia

While low-dose atropine has proven to be effective in slowing myopia progression in children, its administration during the day could impair clear vision due to its mydriatic effects, necessitating nightly dosing to mitigate daytime visual disruption [194]. Furthermore, the long-term use of atropine is associated with potential ocular surface toxicity and a range of side effects [193], making it imperative to consider protective strategies during daylight hours. A novel therapeutic approach could involve the use of a combination eye drop formulation to be administered during the day, containing Lipidure, fucosyl-lactose, and caffeine. Lipidure is a hydrating molecule that serves to protect cellular structures from desiccation and is already utilized in eye drops for dry eye conditions [59]. Fucosyl-lactose has demonstrated protective effects on tear film stability and the ocular surface, particularly in experimental models of atropine-induced dry eye [195]. Caffeine, known for its ability to inhibit chitinases that become activated during ocular surface inflammation (such as in allergic reactions) [196], helps to preserve the epithelial integrity and enhances the tear secretion volume [197]. Additionally, caffeine has been shown to stimulate the synthesis of proteoglycans and collagen in the sclera, potentially reinforcing its structure and thereby slowing the axial elongation associated with myopia progression [198,199]. With pre-existing formulations featuring caffeine displaying good ocular tolerance, this combination could offer a dual benefit: protecting the ocular surface during the day while optimizing the long-term effectiveness of low-dose atropine therapy at night. Ultimately, such an integrated approach could create a more holistic management framework for myopic children, balancing treatment efficacy with comfort and safety.

In addition to this eye drop formulation designed to protect the ocular surface and partially enhance the effects of low-dose atropine administered at night, another promising adjunctive option could be a dietary supplement specifically formulated to improve the efficacy of atropine in controlling myopia.

The integration of a dietary supplement containing 7MX, vitamin D3, and essential amino acids presents a promising approach to addressing myopia progression in children and adolescents. 7MX has shown the ability to stimulate the synthesis of proteoglycans and collagen within the sclera [189,200], a critical factor in maintaining the structural integrity of the eye. Clinical studies involving myopic children have yielded encouraging results, suggesting that this compound can effectively slow myopia progression during these critical developmental years [200].

The role of amino acids—such as glycine, lysine, proline, valine, and alanine—is especially important because they provide metabolic support to the action of 7MX, enhancing its effectiveness in collagen synthesis and overall ocular health. Furthermore, including phenylalanine as a precursor to dopamine is significant, given dopamine’s established role in regulating eye growth and inhibiting excessive elongation of the eyeball [201], which is crucial for children whose visual systems are still developing.

Moreover, vitamin D deficiency has been linked to accelerated myopia progression, potentially through mechanisms involving dopamine [202,203]. Therefore, dietary supplementation with vitamin D3 can help mitigate this risk, contributing to a more stable ocular environment for children as they grow. By combining these ingredients, this dietary supplement not only targets the structural building blocks of the sclera but also addresses the biochemical factors influencing eye growth.

Overall, this integrative approach offers a valuable adjunct to traditional myopia management strategies, enhancing therapeutic outcomes and providing a comprehensive solution for slowing myopia progression in young patients. Given the critical importance of maintaining clear vision during childhood for educational and developmental purposes, implementing this dietary supplement could play a pivotal role in supporting healthy eye development and improving the quality of life for affected children.

#### 5.1.3. The Potential and Limitations of Electrostimulation for Myopia Control

The idea of using electrostimulation to slow myopia progression has been explored in a handful of studies, though the evidence remains limited and somewhat dated. The earliest of these, a 1988 study by Iakimchuk and Verbova [204], tested whether a sinusoidal modulated pulsed current could improve ocular accommodation in school-aged children with progressive myopia. The rationale was that since accommodative dysfunction contributes to myopia development, enhancing this mechanism might help stabilize the refractive error. While the study did report improvements in accommodation, it did not provide conclusive data on whether this actually slowed myopia progression, leaving the broader therapeutic potential uncertain.

A later investigation by Okovitov in 1997 [205] took a more systematic approach, evaluating transconjunctival electroophthalmostimulation (TEOS) in 384 patients with progressive myopia. The results were promising, particularly in mild cases (up to −1.50 D), where participants experienced an average improvement of 0.30 D in visual acuity and a 0.50 D reduction in subjective refraction. The study also noted physiological changes—such as increased rheographic coefficients and an elevated intraocular temperature—suggesting enhanced blood flow and metabolic activity in ocular tissues. Most notably, a long-term follow-up revealed that only 15% of treated patients showed myopia progression over five years, compared to 25% in a control group receiving conventional therapy. These findings imply that electrostimulation may indeed have a stabilizing effect, though the lack of a placebo-controlled design limits definitive conclusions.

Further support came in 2002 from Riabtseva et al. [206], who combined electrostimulation with magnetotherapy and pharmacological treatment in 168 children. Their approach targeted not just accommodation but also underlying metabolic dysfunction, proposing that electrostimulation helped normalize ion transport in ocular tissues and improve ciliary body function. While the study reported positive effects on both accommodation spasms and myopia progression, the use of multiple concurrent therapies makes it difficult to isolate the specific contribution of electrostimulation alone.

Taken together, these studies suggest that electrostimulation could influence myopia progression, primarily through improvements in accommodation and possibly ocular blood flow. However, the evidence remains constrained by methodological limitations—small sample sizes, lack of placebo controls, and the absence of modern biometric measures like axial length tracking. Moreover, the fact that no major studies have revisited this approach in over two decades raises questions about its clinical viability. While the historical data are intriguing, contemporary research with rigorous trial designs would be needed to determine whether electrostimulation deserves a place in modern myopia management strategies.

### 5.2. Presbyopia

Presbyopia is an age-related condition characterized by the gradual loss of the eye’s ability to focus on near objects, typically becoming noticeable after the age of 40. This physiological decline in accommodative capacity arises from two intertwined mechanisms: the progressive weakening of the ciliary muscle (CM) and the increasing rigidity of the crystalline lens [207]. The CM, a key structure in the eye’s accommodative apparatus, contracts to alter the shape of the lens, allowing it to thicken for near vision—a process that diminishes as the muscle atrophies with age [208]. Simultaneously, the lens itself undergoes significant biochemical changes due to age-related factors, primarily the progressive loss of soluble α-crystallin, which is critical for maintaining lens flexibility and counteracting stiffness. This decline is exacerbated by the continuous deposition of lens fibers throughout life, ultimately leading to a marked increase in stiffness and a loss of elasticity, which contributes substantially to the onset and progression of presbyopia [209]. As a result, even when the CM contracts, the hardened lens resists deformation, reducing the eye’s ability to adjust focus efficiently. Together, these age-related changes—muscular degeneration and lens sclerosis—compromise the dynamic interplay required for accommodation, culminating in the need for corrective lenses to restore near vision.

Therefore, given the respective roles played by the CM and the lens in the onset and progression of presbyopia, interventions aimed at strengthening the CM and softening the lens should be able to improve this annoying refractive defect of old age.

#### 5.2.1. The Potential and Limitations of Electrostimulation for Presbyopia Control

We have explored the role of the ciliary muscle in presbyopia, investigating whether enhancing its contractile function could partially compensate for the progressive stiffening of the crystalline lens. Our innovative approach demonstrated that targeted ciliary muscle electrostimulation (ES) can partially restore the accommodative function in early presbyopia [210]. In this controlled clinical study, we applied pulsed microcurrents (26 mA, 2 s on/6 s off) via scleral lenses to stimulate the ciliary muscle. After just four treatment sessions, participants showed statistically significant improvements in uncorrected near visual acuity (UNVA) and reading speed. Ultrasound biomicroscopy provided objective confirmation, revealing an increased lens thickness (+0.07 mm) and altered curvature during accommodation attempts—findings consistent with restored ciliary muscle efficacy. While Helmholtz’s theory of lens hardening remains central to presbyopia pathophysiology, our results demonstrate that the ciliary muscle retains measurable plasticity. The study’s robust design (age-matched cohorts) and high patient satisfaction (66.7% reporting being “very satisfied”) support the concept that presbyopia involves not only structural changes but also reversible functional decline. However, the transient nature of improvements—requiring periodic ES sessions—suggests that sustained stimulation is needed to counteract progressive biomechanical losses. These findings reposition the ciliary muscle as a modifiable therapeutic target in early presbyopia. Though lens rigidity remains the primary pathological driver, our work indicates that ciliary muscle revitalization through ES, pharmacotherapy, or combined approaches could expand treatment paradigms for this condition.

#### 5.2.2. Emerging Pharmacological Approaches for Presbyopia Management

While electrostimulation (ES) offers a mechanical approach to restoring ciliary muscle contractility, complementary biochemical strategies may address the metabolic and structural components of presbyopia. L-Carnitine emerges as a promising therapeutic candidate due to its dual effects on both the crystalline lens and ciliary muscle—key structures in ocular accommodation.

The age-related decline in soluble α-crystallin, a critical lens protein maintaining flexibility, contributes significantly to presbyopia development [209]. Research demonstrates that L-carnitine preserves α-crystallin’s molecular chaperone activity by reducing oxidative damage and preventing post-translational modifications that lead to protein denaturation [211]. This protective effect may help maintain lens pliability and delay presbyopia onset by sustaining adequate levels of functional α-crystallin.

Beyond lens effects, L-carnitine enhances ciliary muscle performance through improved bioenergetics. As shown in muscle metabolism studies [212], L-carnitine optimizes mitochondrial functions and cellular energy production. In the context of presbyopia, this metabolic support could increase the ciliary muscle’s contractile endurance—particularly important as the lens loses natural elasticity with age.

This dual mechanism positions L-carnitine as a comprehensive intervention for presbyopia: preserving lens flexibility through α-crystallin stabilization while enhancing ciliary muscle function via improved energy metabolism. Such combined action addresses both the structural and functional aspects of accommodation loss. Further research should explore optimal dosing regimens and potential synergies with mechanical approaches like ES [210] to develop effective presbyopia management strategies.

Recent advances in ocular pharmacology have revealed several promising compounds that target the fundamental mechanisms of presbyopia. Among these, lipoic acid has shown particular potential when administered as an eye drop, with clinical trials demonstrating its ability to improve near visual acuity by enhancing lens flexibility [213]. Similarly, preclinical studies with 25-hydroxycholesterol indicate that this compound can modify lens viscoelastic properties through distinct biochemical pathways [214]. Beyond topical applications, orally administered α-glucosyl-hesperidin has emerged as a systemic alternative, with research suggesting that it may influence lens sclerosis while improving accommodative ability [215]. These diverse pharmacological strategies collectively address the multifaceted nature of age-related lens stiffening, offering complementary approaches to mechanical interventions like electrostimulation.

The binocular eye drop approach represents an innovative pharmacological strategy that differs fundamentally from traditional treatments. Developed by Renna and colleagues [216], this method involves specially formulated drops administered to both eyes that combine multiple active compounds: parasympathetic agents to stimulate ciliary muscle contraction, NSAIDs to modulate inflammatory pathways, and alpha-agonists to fine-tune the pupillary response. Unlike conventional eye drops that often cause blurred distance vision when improving near focus, this carefully balanced formulation aims to enhance reading vision while preserving distance acuity. The treatment regimen of twice-daily application appears to achieve this delicate balance by pharmacologically activating the iris and ciliary muscle in a coordinated manner, mimicking natural accommodation while minimizing visual side effects.

Building on these developments, a comprehensive treatment paradigm could integrate multiple delivery routes for synergistic effects. A topical formulation combining lipoic acid with L-carnitine might target both lens elasticity and ciliary muscle metabolism [213,217], while an oral supplement containing L-carnitine and α-glucosyl-hesperidin could provide systemic support [215,218]. When used in conjunction with the binocular eye drop approach [216], this multimodal strategy could potentially prime the ocular system for enhanced responsiveness to electrostimulation while extending the therapeutic benefits between treatments.

The scientific rationale for such combined therapy lies in addressing presbyopia’s complex pathophysiology through complementary mechanisms: improving lens biomechanics, enhancing ciliary muscle function, and optimizing pupillary dynamics. This integrated approach represents a significant evolution from single-target interventions, potentially offering more sustained and naturalistic vision correction. While further clinical validation is needed, these pharmacological advances suggest a future where presbyopia management may shift from simple optical correction to comprehensive functional restoration of the accommodative system.

## 6. Integrated Strategies for Visual Rehabilitation: Multisensory and Optogenetic Approaches

The interplay between multisensory integration and optogenetic technologies represents a transformative frontier in visual rehabilitation, offering innovative solutions to restore function in individuals with visual impairments. These approaches, though distinct in methodology, share a common goal: harnessing the brain’s remarkable plasticity to compensate for lost or degraded vision. By combining insights from comparative neurobiology and cutting-edge biomedical engineering, I present here possible interventions that not only bypass damaged neural pathways but also amplify residual sensory processing through cross-modal reinforcement.

**Figure 4:**
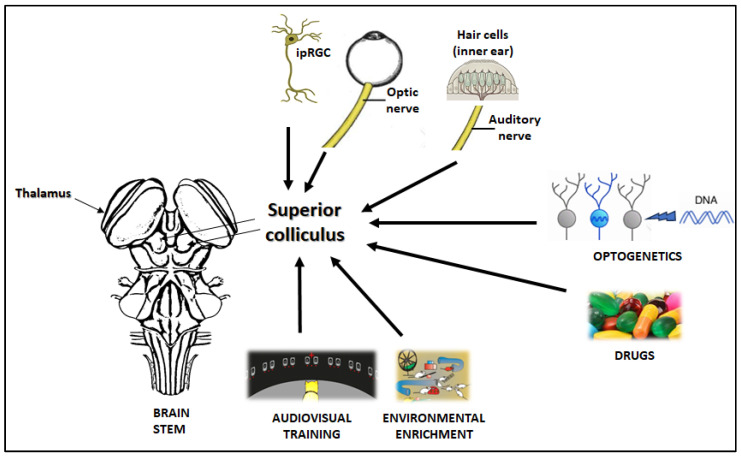
Visual rehabilitation strategies. As the primary integration hub for multisensory inputs—including retinal signals from retinal ganglion cells (RGCs) and intrinsically photosensitive RGCs (ipRGCs), as well as auditory pathways (illustrated in the upper panel)—the superior colliculus (SC) plays a pivotal role in visual rehabilitation. Audiovisual training (e.g., AViST) and environmental enrichment enhance plasticity by leveraging the SC’s multisensory integration capabilities. Complementary approaches, such as optogenetic stimulation and pharmacological adjuvants (e.g., D-cycloserine, fluoxetine), can further potentiate SC function and amplify training efficacy.

At the core of multisensory rehabilitation lies the superior colliculus (SC) in mammals and its evolutionary counterpart, the optic tectum in non-mammalian species (Figure 4). These midbrain structures serve as critical hubs for integrating visual, auditory, and somatosensory inputs, enabling organisms to navigate complex environments [219]. In cases of visual impairment, the SC exhibits remarkable adaptability, shifting its reliance toward intact sensory modalities to maintain spatial awareness and orienting behaviors. For instance, auditory cues can enhance the detection of visual stimuli in the blind field of hemianopic patients, a phenomenon leveraged by rehabilitation protocols such as Audio-Visual Scanning Training (AViST). This technique pairs spatially and temporally congruent auditory and visual stimuli to strengthen residual subcortical pathways, particularly the retino–colliculo–extrastriate route, which remains functional even after primary visual cortex damage [220].

Complementing these natural compensatory mechanisms, optogenetic strategies provide a direct means to restore neural activity in dysfunctional visual circuits. By introducing light-sensitive proteins like channel rhodopsin into surviving retinal ganglion cells (RGCs) or higher-order visual areas, researchers can artificially recreate light-evoked signaling in the absence of functional photoreceptors [220]. Pioneering clinical work has demonstrated the potential of this approach: in a landmark trial, a blind patient expressing ChrimsonR in RGCs regained partial light perception after training with engineered goggles that converted visual scenes into optogenetic stimuli [221]. Beyond the retina, optogenetic stimulation of the SC or lateral geniculate nucleus (LGN) in animal models generates artificial percepts, suggesting that bypassing early visual pathways may still yield meaningful visual experiences [219].

Environmental enrichment (EE) further enhances neuroplasticity in visual rehabilitation by providing structured, multisensory stimulation that promotes the adaptive rewiring of neural circuits [222]. Studies in animal models demonstrate that EE—combining physical activity, social interaction, and sensory–motor challenges—upregulates neurotrophic factors like BDNF and enhances synaptic plasticity in visual and multisensory areas [223]. In humans, enriched rehabilitation settings that incorporate tactile exploration, auditory feedback, and navigational tasks can improve spatial cognition and compensatory visual behaviors, particularly when combined with targeted therapies such as AViST [224]. Multimodal feedback systems (e.g., virtual reality) leverage cross-modal interactions to reinforce sensorimotor integration, a principle adaptable to visual rehabilitation. For instance, synchronized visual–tactile stimuli enhance cortical activation in sensory-deprived patients, suggesting that similar strategies could optimize compensatory visual pathways [224,225]. The dynamic interplay between EE and optogenetic interventions is an emerging area of interest, as enriched environments may prime neural circuits for the more effective integration of artificial visual inputs [220].

The synergy between these approaches becomes particularly compelling when considering their combined application. Multisensory training primes the brain to integrate cross-modal inputs, while optogenetics provides a tool to precisely activate specific neural populations. For example, pairing auditory cues with optogenetic stimulation of the SC could reinforce spatial maps in visually impaired individuals, enhancing their ability to localize objects. Pharmacological adjuvants may further optimize outcomes: drugs like D-cycloserine, which potentiates NMDA-receptor-dependent plasticity, could accelerate the learning of multisensory associations, while fluoxetine, by boosting BDNF levels, might sustain long-term synaptic reorganization [220].

Looking ahead, key challenges must be addressed to translate these strategies into widespread clinical use. Optimizing opsin delivery methods, minimizing immune responses to viral vectors, and personalizing multisensory training protocols will be critical. Comparative studies across species—from birds with highly developed tectofugal pathways to mammals with cortical-dominated vision—could uncover conserved plasticity mechanisms ripe for therapeutic exploitation [219]. Additionally, the role of intrinsically photosensitive RGCs (ipRGCs) in non-image-forming vision warrants further exploration, as these cells may provide a parallel pathway for light detection in degenerative blindness [226].

In summary, the integration of multisensory rehabilitation and optogenetics offers a dual-path framework for visual restoration. Where multisensory training capitalizes on the brain’s innate ability to reweight sensory inputs, optogenetics provides a tool to artificially resurrect neural signaling. Together, they exemplify the power of bridging fundamental neuroscience with translational innovation—a paradigm that may ultimately redefine the boundaries of visual rehabilitation.

## 7. Emerging Frontiers in Ophthalmic Therapeutics

The management of ophthalmic pathologies has undergone significant advancements over the past two decades, driven by innovative research and rapid advancements in pharmacology, biotechnology, and neuro-engineering. As we conclude this comprehensive exploration of ophthalmic pathologies and their management, it is imperative to highlight the most recent innovations that promise to redefine therapeutic paradigms. These developments not only address longstanding challenges in drug delivery, neuroprotection, and sensory restoration but also underscore the importance of personalized and multimodal approaches in treating complex ocular diseases.

### 7.1. Next-Generation Drug Delivery Systems

Recent advancements in ocular drug delivery have prioritized innovative strategies to bypass the eye’s complex anatomical and physiological barriers, thereby optimizing therapeutic outcomes. Nanotechnology-driven formulations—such as lipid nanocapsules, polymeric micelles, and other nanosized carriers—have demonstrated significant potential in enhancing the bioavailability of poorly soluble therapeutics, including EGCG and melatonin. These systems facilitate sustained drug release, reduce the administration frequency, and mitigate systemic adverse effects, addressing critical challenges in managing chronic ocular pathologies like glaucoma and AMD [7,227].

However, some relevant challenges still persist, because the cornea and blood–retinal barrier limit passive diffusion, requiring targeted designs (e.g., ligand-conjugated nanoparticles) [7]. Manufacturing complexity and batch-to-batch variability hinder the largescale production of nanocarriers [8]. Potential nanoparticle accumulation in ocular tissues warrants extended toxicological studies [227].

Gene therapy has also transitioned from experimental models to clinical reality, with adeno-associated virus (AAV) vectors delivering neuroprotective genes (e.g., BDNF, CNTF) directly to RGCs in glaucoma [228]. Sharif [229] underscores the dual challenge of enhancing the trabecular meshwork (TM)/Schlemm’s canal (SC) outflow while preserving RGCs, advocating for combinatorial approaches that pair IOP-lowering drugs (e.g., rho kinase inhibitors like netarsudil) with neuroprotective gene therapies [230]. Moreover, CRISPR-based interventions are being explored for inherited retinopathies, offering the potential for precise genomic corrections [229]. Beyond gene therapy, other pharmacological approaches, such as glucagon-like peptide-1 receptor agonists (GLP-1 RAs), are being explored for their potential benefits in retinal diseases, including diabetic retinopathy [231]. Recent work by Mohanty et al. [232] exemplifies innovative gene therapy approaches for glaucoma, introducing an engineered mechanosensitive channel (SAM) delivered via AAV vectors to trabecular meshwork cells. This “barogenetic” technology acts as a pressure-sensitive release valve, lowering the intraocular pressure (IOP) by enhancing aqueous humor outflow, offering a targeted alternative to traditional pharmacological or surgical interventions. Despite advancements, Sharif [229] notes that sustained IOP control remains incomplete in many patients due to TM/SC pathway occlusion, necessitating novel devices (e.g., Preserflo^®^ microshunt) or dual-action drugs like latanoprostene bunod (NO-donor + prostaglandin analog).

Gene therapy has achieved milestones, as described above but faces immune response issues, because preexisting immunity to AAV vectors may limit efficacy [228]. Durability issues, because transient gene expression necessitates repeat administrations in chronic conditions.

### 7.2. Neuroprotection and Metabolic Modulation

The recognition of glaucoma and other optic neuropathies as neurodegenerative disorders has spurred interest in therapies targeting mitochondrial dysfunction and oxidative stress. Sharif [229] emphasizes that neuroprotection must address both mechanical stress from elevated IOP and inflammatory neurodegeneration, proposing the adjunctive use of cytoprotective agents (e.g., brimonidine) alongside IOP-lowering drugs. Nicotinamide riboside (NR), a precursor to NAD+, has emerged as a promising adjunct therapy for RGC preservation in glaucoma, potentially by replenishing NAD+ levels to support mitochondrial function and mitigate oxidative stress [233]. Sharif [234] highlights complementary strategies for RGC preservation, including gene therapies targeting oxidative stress and mitochondrial dysfunction in glaucoma, underscoring the need to pair IOP-lowering therapies with neuroprotective agents. Similarly, electrical and electromagnetic stimulation (e.g., transcranial magnetic therapy) have shown promise in preclinical models for enhancing RGC survival and axonal regeneration, though clinical translation remains nascent [235]. Sharif further highlights the limitations of current neuroprotective strategies, including the poor blood–retinal barrier penetration of systemic agents (e.g., NR) and the need for localized delivery systems to enhance retinal bioavailability [229].

Intriguingly, the repurposing of GLP1 receptor agonists (e.g., semaglutide) for diabetic retinopathy (DR) remains controversial, with some studies suggesting potential benefits for metabolic and ocular health—such as reduced systemic inflammation and macrovascular risk—while others report the early worsening of DR after initiation. Although preclinical data suggest mechanisms by which these drugs might mitigate retinal inflammation or vascular leakage, clinical evidence is inconclusive, and ophthalmologic monitoring is prioritized [236]. While metabolic modulators like GLP-1 agonists show potential, Sharif [229] cautions that their systemic effects may not directly translate to optic nerve protection, urging targeted therapies to mitigate glaucomatous neurodegeneration independent of glycemic control.

Nicotinamide riboside (NR) may show promise for preserving RGCs by boosting NAD+ levels [233], yet systemic NR administration achieves low ocular concentrations, demanding localized delivery methods. Moreover, the precise pathways linking NAD+ depletion to glaucoma progression remain unclear [233]. GLP1 receptor agonists (e.g., semaglutide) are debated for diabetic retinopathy due to paradoxical effects, because the early worsening of retinopathy has been observed in some patients, possibly tied to rapid glycemic control [236]. Moreover, a limited-ocular-penetration issue might exist, if systemic delivery does not reach therapeutic retinal levels.

### 7.3. Bioprinting and Regenerative Medicine

The advent of 3D bioprinting has opened new avenues for corneal repair, with bioengineered grafts seeded with patient-derived limbal stem cells emerging as a promising alternative to donor tissues. While preclinical studies demonstrate the feasibility of 3D-printed corneal layers using stem cell-laden bioinks, clinical translation remains in the early stages, focusing on addressing donor shortages and improving graft integration [237]. The scaffold-based transplantation of photoreceptor precursors has emerged as a promising therapeutic strategy for retinal degenerative diseases like retinitis pigmentosa. Preclinical studies report improved light sensitivity in animal models through enhanced donor cell integration and material transfer, though challenges in cell maturation and functional outcomes persist [238,239].

3D-bioprinted corneal grafts indeed address donor shortages [237], but are confronted with cell viability issues, in order to maintain stem cell potency during printing and post-implantation [237]. Integration issues ensure graft transparency and neural reinnervation for functional recovery.

On the other end, photoreceptor precursor transplantation for retinal degeneration [238] struggles with engraftment efficiency: <5% of transplanted cells typically integrate into the host retina [239]. Functional outcomes are because of limited evidence of restored vision in clinical trials.

### 7.4. Artificial Intelligence and Precision Medicine

AI-driven diagnostics are transforming early disease detection in ophthalmology. Deep learning algorithms trained on optical coherence tomography (OCT) datasets have demonstrated potential to identify early signs of glaucoma and other eye diseases, though clinical validation for long-term progression prediction remains ongoing [240]. Meanwhile, wearable devices equipped with real-time intraocular pressure (IOP) monitoring enable dynamic adjustments in glaucoma therapy, moving beyond static office measurements [241]. AI tools may further benefit from integrating genetic risk data (e.g., GWAS findings for glaucoma subtypes) to refine personalized treatment plans, as emphasized by Sharif [234].

However, AI algorithms for OCT-based glaucoma detection [240] face issues of data bias, because models trained on homogeneous populations may fail in diverse cohorts. Clinical adoption problems are because of a lack of FDA-cleared AI tools for real-time decision support.

Wearable IOP sensors [241] are limited by accuracy, since dynamic IOP fluctuations complicate calibration against gold-standard tonometry.

### 7.5. Optogenetics and Sensory Substitution

Beyond the landmark success of optogenetic vision restoration in retinitis pigmentosa [221], engineered opsins with enhanced light sensitivity (e.g., ChrimsonR, MCO1) are broadening the therapeutic potential for retinal degenerations. While newer variants like redshifted opsins improve safety and temporal resolution, challenges persist in achieving functional vision under ambient light without auxiliary devices [242]. Concurrently, noninvasive sensory substitution devices—such as auditory-to-visual neural implants—are being refined to improve spatial navigation in blind patients [243].

Yet, optogenetic vision restoration (e.g., ChrimsonR in retinitis pigmentosa [221]) grapples with light sensitivity issues, since engineered opsins require intense light stimulation, limiting ambient-light utility [242]. Immune rejection problems occur due to the fact that the long-term stability of viral vector-transduced cells remains unproven. Notably, optogenetic approaches for RGC protection (e.g., light-sensitive ion channels) are being explored alongside vision-restoration strategies, though both face similar hurdles in achieving functional outcomes [234].

### 7.6. Future Directions and Author’s Perspective

As we reflect on twenty-five years of progress in ophthalmic research, the path forward presents both unprecedented opportunities and formidable challenges. The transition from symptomatic treatment to mechanism-based therapies has already begun to reshape clinical practice, yet significant work remains to fully realize this paradigm shift.

The development of combinatorial approaches represents one of the most promising avenues for complex ocular diseases. In diabetic retinopathy, for instance, pairing anti-VEGF agents with anti-inflammatory treatments could address multiple pathological pathways simultaneously [244]. Similarly, the neuroprotective strategies discussed in this review, particularly those targeting mitochondrial dysfunction and oxidative stress, may soon complement traditional IOP-lowering therapies in glaucoma management [233]. Combinatorial strategies could extend to pairing SAM-based IOP modulation [232] with neuroprotective agents (e.g., NR or electrical stimulation [235]) to address both mechanical and metabolic insults in glaucoma. Combinatorial approaches represent a promising avenue for complex ocular diseases. Sharif [229] advocates for “vertical” therapeutic strategies that simultaneously target aqueous humor dynamics (e.g., via EP2 agonists like omidenepag isopropyl) and RGC survival pathways (e.g., BDNF gene therapy), addressing both the upstream and downstream pathology in glaucoma.

Technological innovations continue to push the boundaries of what is possible in ocular therapeutics. Nanotechnology platforms have demonstrated remarkable potential for enhancing drug delivery, as evidenced by recent work with melatonin nanomicelles and lipid-based formulations [121,227]. Meanwhile, advances in bioprinting are bringing us closer to solving the critical shortage of donor corneas, with early studies showing promising results for 3D-printed grafts [237]. Microbiome modulation via probiotic bioprinting might bring new treatment strategies for ocular surface dysbiosis linked to dry eye disease [74]. Similarly, noninvasive neuromodulation (e.g., electroacupuncture) may augment traditional therapies by promoting RGC resilience, though rigorous clinical validation is needed [235]. Neural prosthetics interfacing with visual cortex implants might lead to bypassing damaged optic pathways [245].

Perhaps most exciting are the emerging opportunities in personalized medicine. The theranostic approach to corneal crosslinking, which allows the real-time monitoring of treatment efficacy, exemplifies how biomarker-guided strategies can optimize outcomes [66,67]. When combined with AI-driven diagnostic tools, these techniques could enable truly individualized treatment plans tailored to each patient’s unique disease profile.

However, several critical challenges must be addressed to translate these innovations into widespread clinical practice. Drug delivery remains a persistent obstacle, particularly for posterior segment diseases where the blood–retinal barrier limits therapeutic access [7]. The development of novel delivery systems, including sustained-release implants and nanoparticle formulations, will be essential to overcome these anatomical barriers [16,227].

Global accessibility presents another significant hurdle. While cutting-edge therapies like optogenetic vision restoration show remarkable potential [221], their high costs may limit availability in resource-limited settings. This underscores the importance of developing affordable alternatives, such as the nutraceutical formulations discussed in this review [156,160]. As Sharif notes, the high cost of advanced therapies (e.g., microshunts, gene editing) risks exacerbating global disparities in glaucoma care, underscoring the need for affordable neuroprotective nutraceuticals or repurposed drugs [229].

Looking ahead, the author envisions an ophthalmic landscape where early intervention and prevention take precedence over late-stage disease management. The success of low-dose atropine in slowing myopia progression demonstrates the tremendous value of preventive approaches [183,184,185,186]. Similar strategies could be applied to other ocular conditions, potentially reducing the global burden of vision loss.

Ultimately, realizing this vision will require unprecedented collaboration across disciplines. Engineers, basic scientists, and clinicians must work together to bridge the gap between bench and bedside. With sustained investment and innovation, the coming decades may witness transformative advances that redefine our approach to ocular health and vision preservation.

## 8. Conclusions

This subjective overview synthesizes a comprehensive body of work aimed at addressing the limitations of current therapies for ocular surface diseases, glaucoma, retinopathies, and refractive errors. Key contributions include the identification of natural compounds, such as epigallocatechin gallate (EGCG) and forskolin, which offer neuroprotective and intraocular-pressure-lowering benefits, and the development of novel drug molecules like Uparant for retinal neovascularization. Additionally, advancements in drug delivery systems, such as nanomicellar and liposomal technologies, have enhanced the efficacy and tolerability of treatments for conditions like dry eye syndrome and keratoconus.

The exploration of visual rehabilitation strategies, including multisensory integration and optogenetic approaches, represents a paradigm shift in restoring functions for individuals with irreversible vision loss. By leveraging the brain’s plasticity and combining sensory training with cutting-edge biomedical engineering, these methods offer hope for improved quality of life in visually impaired patients. Furthermore, the integration of dietary supplements and personalized medicine underscores the importance of holistic and targeted approaches in ophthalmology.

Despite these advancements, challenges remain in optimizing therapeutic protocols, ensuring long-term safety, and translating preclinical findings into clinical practice. Future research should focus on refining these innovations, exploring synergistic combinations, and addressing unmet needs in ocular health. By continuing to bridge the gap between scientific discovery and clinical application, we can pave the way for more effective, patient-centered solutions in the prevention and treatment of vision-threatening diseases. This collective effort not only enhances our understanding of ocular pathologies but also reaffirms the potential of interdisciplinary collaboration to transform the landscape of eye care.

## Figures and Tables

**Table 1:** Food supplements for ocular health. Paradigmatic molecules in food supplements that have demonstrated efficacy in combating eye diseases.

Active Principle	Description	Effects	References
Forskolin	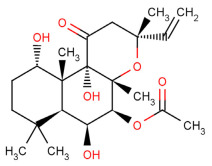	Decreases IOP (indirect neuroprotection); Stimulates BDNF signaling (direct neuroprotection).	[94,95,96,97,98,99,100,101,102,103,104,105,106,107]
Citicoline	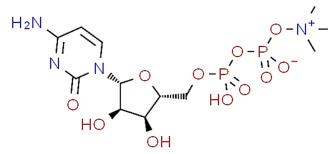	Enhances mitochondrial energy production; Reduces oxidative stress; Restores mitochondrial membrane potential; Improves mitochondrial biogenesis.	[109,110,111,112,113,118]
Fatty acids	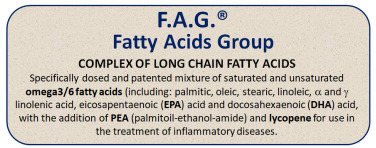	Trigger the shift of macrophages from the M1 pro-inflammatory state to the M2 anti-inflammatory state.	[146,147,148]
Antioxidants	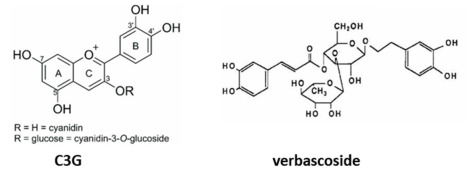	Decrease oxidative stress and inflammation improving visual acuity.	[149,150,151,152,153]

## Data Availability

No new data were created or analyzed in this study. Data sharing is not applicable.
